# Metatranscriptomic Analysis of the Mouse Gut Microbiome Response to the Persistent Organic Pollutant 2,3,7,8-Tetrachlorodibenzofuran

**DOI:** 10.3390/metabo10010001

**Published:** 2019-12-18

**Authors:** Robert G. Nichols, Jingtao Zhang, Jingwei Cai, Iain A. Murray, Imhoi Koo, Philip B. Smith, Gary H. Perdew, Andrew D. Patterson

**Affiliations:** 1 Center for Molecular Toxicology and Carcinogenesis, Department of Veterinary and Biomedical Sciences, The Pennsylvania State University, University Park, PA 16802, USA; rgn5011@psu.edu (R.G.N.); jingtao.zhang@ntu.edu.sg (J.Z.); vivianna620@gmail.com (J.C.); iam1@psu.edu (I.A.M.); iuk41@psu.edu (I.K.); ghp2@psu.edu (G.H.P.); 2 Metabolomics Facility, Huck Institutes of the Life Sciences, The Pennsylvania State University, University Park, PA 16802, USA; pbs13@psu.edu

**Keywords:** Ah receptor, TCDF, gut microbiome, metatranscriptomics, lipopolysaccharide (LPS)

## Abstract

Persistent organic pollutants (POPs) are important environmental chemicals and continued study of their mechanism of action remains a high priority. POPs, such as 2,3,7,8-tetrachlorodibenzo-p-dioxin (TCDD), 2,3,7,8-tetrachlorodibenzofuran (TCDF), and polychlorinated biphenyls (PCBs), are widespread environmental contaminants that are agonists for the aryl hydrocarbon receptor (AHR). Activation of the AHR modulates the gut microbiome community structure and function, host immunity, and the host metabolome. In the current study, male C57BL6/J mice were exposed, via the diet, to 5 µg/kg body weight (BW) TCDF or 24 µg/kg BW of TCDF every day for 5 days. The functional and structural changes imparted by TCDF exposure to the gut microbiome and host metabolome were explored via 16S rRNA gene amplicon sequencing, metabolomics, and bacterial metatranscriptomics. Significant changes included increases in lipopolysaccharide (LPS) biosynthesis gene expression after exposure to 24 µg/kg BW of TCDF. Increases in LPS biosynthesis were confirmed with metabolomics and LPS assays using serum obtained from TCDF-treated mice. Significant increases in gene expression within aspartate and glutamate metabolism were noted after exposure to 24 µg/kg BW of TCDF. Together, these results suggest that after exposure to 24 µg/kg BW of TCDF, the gut microbiome increases the production of LPS and glutamate to promote localized gut inflammation, potentially using glutamate as a stress response.

## 1. Introduction

The relationship between the intestinal microbiome and the host metabolome is important to understand drug and xenobiotic metabolism, and mode of action [1,2]. Intestinal bacteria express enzymes, like β-glucuronidases, that may activate (and sometimes reactivate) certain drugs [3], and intestinal bacteria can alter how the host metabolizes drugs and xenobiotics [4,5]. Changes in the composition of the intestinal bacteria can influence the host due to the role intestinal microbiota play in drug metabolism [6], food metabolism [7], and gut barrier function [8].

2,3,7,8-tetrachlorodibenzo-*p*-dioxin (TCDD), along with 2,3,7,8-tetrachlorodibenzofuran (TCDF) and coplanar polychlorinated biphenyls (PCBs), are agonists of the aryl hydrocarbon receptor (AHR) and have been reported to modulate the intestinal microbiome [9,10,11,12]. The AHR plays important roles in both adaptive and innate immunity (pro-inflammatory and anti-inflammatory responses) and in gut barrier function [13,14]. AHR activity can be modulated by a variety of tryptophan metabolites produced by the host and the microbiota [15]. The AHR induces the expression of several cytochrome P450 genes, including *CYP1A1*, which is important for the metabolism of numerous xenobiotics [13]. Since AHR is also a regulator of intestinal immune homeostasis, chronic activation (e.g., from environmental pollutants) can impact the microbiome composition [9,16].

AHR-dependent changes in the microbiome can cause increased gut inflammation, which can lead to further intestinal dysfunction, thus worsening inflammation [17,18]. A major contributor to gut inflammation is lipopolysaccharide (LPS) [19]. LPS is located in the outer membrane of gram-negative bacteria and can be released from gram-negative bacteria that are killed by host defenses. Additionally, gram-negative bacteria can escape the gut via a leaky gut barrier, which may be associated with increased gut inflammation and disorder of the intestinal microbiome [8], which can exacerbate metabolic disorders like obesity and diabetes [9,16].

TCDF is an AHR agonist with reduced potency and half-life in vivo relative to TCDD but has a similar mode of action [20,21]. In this study, C57Bl6/J male mice were treated via the diet with 5 µg/kg body weight (BW) or 24 µg/kg BW of TCDF every day for 5 days. Previously, 24 µg/kg BW of TCDF has been validated with allometric calculations to represent a dose of 3000 ng/kg in humans, in mice [9]. Moreover, 3000 ng/kg represented the exposure level of the Seveso, Italy 2,4,5-trichlorophenol reactor accident, where human exposures were reported to be between 2.5 and 56,000 ng/kg in blood [22]. Liquid chromatography coupled with mass spectrometry (LCMS), endotoxin assays, 16S rRNA gene amplicon sequencing, pathway enrichment analysis, and bacterial metatranscriptomics were used to investigate and validate TCDF modulation of the intestinal microbiome composition and function. Together these experiments identified significant increases in LPS production pathways, bacterial stress responses (possibly through glutamate utilization), and circulating LPS in mouse serum, which provide further evidence of the importance of the interaction between the microbiome and the host in mediating the effects of toxicants like TCDF.

## 2. Results and Discussion

### 2.1. Bacterial Taxonomic Shifts After TCDF Treatment

**Figure 1:**
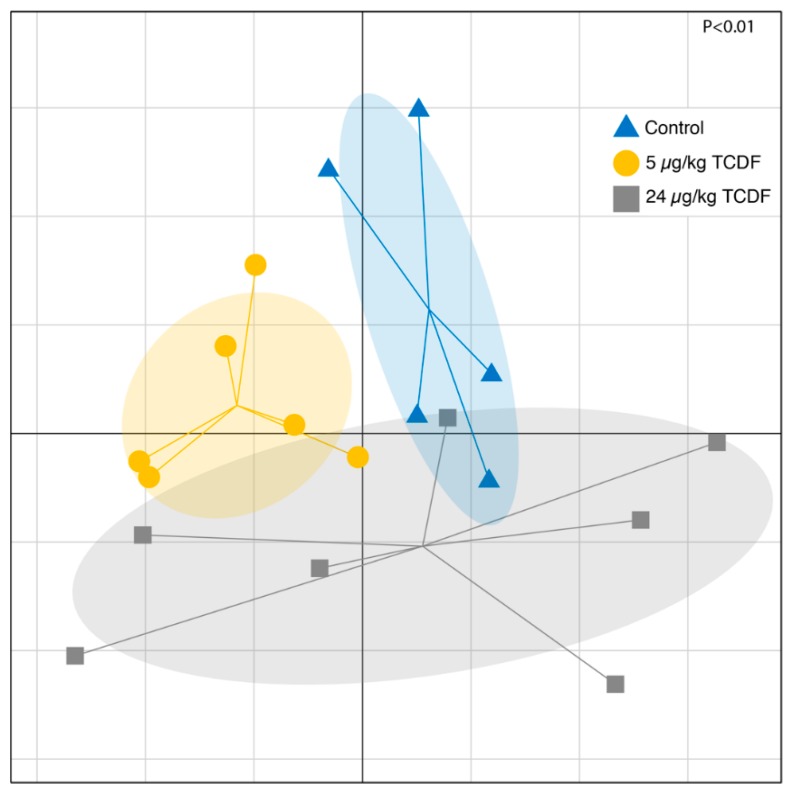
Bacterial taxonomic shifts after dietary exposure to 5 or 24 µg/kg BW TCDF. Variance-adjusted weighted UniFrac analysis of the beta diversity after treatment of 5 µg/kg BW TCDF (gold) or 24 µg/kg BW TCDF (grey) in relation to the control (blue). *p* values were determined via permutational multivariate analysis of variance using the Adonis function from the vegan package in R studio.

**Figure 2:**
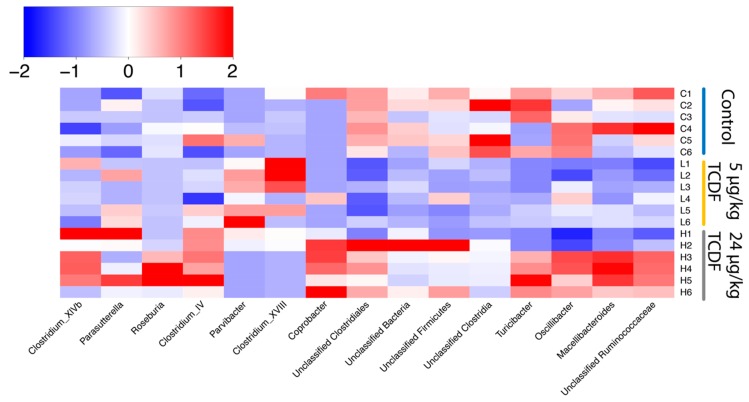
Significant genus level changes after 5 or 24 µg/kg BW TCDF exposure. Significantly different genera between 5 µg/kg and 24 µg/kg BW TCDF, and the control (*p* < 0.05). Data were z-scored according to the formula $\frac{\left(x-\bar{x}\right)}{sd\left(x\right)}$ (*n* = 6 per group).

Bacterial population shifts seen in Figure 1 were significant after permutational multivariate ANOVA using distance matrices (R function Adonis in the vegan package) and are illustrated using Unifrac distances. The differential effects on the microbiome from either 5 µg/kg BW TCDF or 24 µg/kg BW TCDF exposures support the importance of balanced AHR activity to maintain the microbiome community [10]. Additionally, a heat map of z-scores created from all significantly different bacteria genera after 5 and 24 µg/kg BW TCDF is shown in Figure 2. Of particular note are *Clostridium* clusters XIVb and IV, which were both significantly decreased in the 24 µg/kg BW TCDF mice. Additionally, Figure 2 demonstrates significant decreases of *Oscilibacter* and unclassified *Ruminococcaceae* in the 5 µg/kg BW TCDF, but these changes were not significant following 24 µg/kg BW TCDF exposure. Phyla shifts were not significant; however, trends seen in Figure S1 match what has been previously reported [9,23]. Additionally, according to the mock sample included in the sequence run, the error rate for the sequencing run was ~1%, which is higher than the Illumina reported error rate of 0.1% [24].

### 2.2. PICRUSt Functional Predictions

PICRUSt (Phylogenic Investigation of Communities by Reconstruction of Unobserved States) predicts the functional changes of the microbiome by examining taxonomical shifts in the microbiome and assigning known functions to the intestinal bacteria that increased or decreased in abundance. PICRUSt predictions are based on the functional capability of the microbiome rather than a direct examination of actively transcribed genes. After PICRUSt, 14 different pathways were predicted to be significantly upregulated or significantly downregulated after the 24 µg/kg BW TCDF treatment (Table 1). Of particular note, valine, leucine, and isoleucine degradation and pathways with unknown function were predicted to be upregulated after the 24 µg/kg BW TCDF treatment. PICRUSt predicted that amino sugar and nucleotide sugar metabolism, lysine biosynthesis, arginine and proline metabolism, and starch and sucrose metabolism should be downregulated after the 24 µg/kg BW TCDF treatment. There were no predicted changes after the 5 µg/kg BW TCDF treatment.

### 2.3. Shifts in the Metabolome after TCDF Treatment

Table 2 illustrates the 33 significant metabolite changes out the 147 total identified metabolites. The 147 metabolites that were identified after LC-MS analysis are reported in Table S2. Over 30,000 unidentified features were captured and the top 200 significant unidentified features are reported in Table S3. Amino acids like asparagine (2.86 times higher after 24 µg/kg BW exposure to TCDF, *p* = 0.04), glycine (1.41 times higher after 24 µg/kg BW exposure to TCDF, *p* = 0.02), proline (1.47 times higher after 24 µg/kg BW exposure to TCDF, *p* = 0.04), serine (1.57 times higher after 24 µg/kg BW exposure to TCDF, *p* = 0.01), leucine (1.81 times higher after 24 µg/kg BW exposure to TCDF, *p* = 0.003), threonine (1.42 times higher after 24 µg/kg BW exposure to TCDF, *p* = 0.004), and tryptophan (1.60 times higher after 24 µg/kg BW exposure to TCDF, *p* = 0.009) significantly increased after the 24 µg/kg BW exposure to TCDF. Additionally, there were significant decreases in UDP-N-acetyl-glucosamine (0.3 times lower after 5 µg/kg BW exposure to TCDF, *p* = 0.019) and N-Acetyl-glucosamine-1/6-phosphate (0.16 times lower after 24 µg/kg BW exposure to TCDF, *p* = 0.002) in the 5 and 24 µg/kg BW exposure to TCDF, respectively.

All metabolites identified were then used for pathway enrichment through MetaboAnalyst [26,27,28]. The significantly enriched pathways when comparing the control with the mice treated with 24 µg/kg BW TCDF are reported in Table 3. No significant pathways were enriched when comparing the control with the mice treated with 5 µg/kg BW TCDF. Of particular note, glycine, serine, and threonine metabolism, and phenylalanine, tyrosine, tryptophan, valine, leucine, and isoleucine biosynthesis were all enriched.

### 2.4. Changes in Overall Microbiome Function

**Figure 3:**
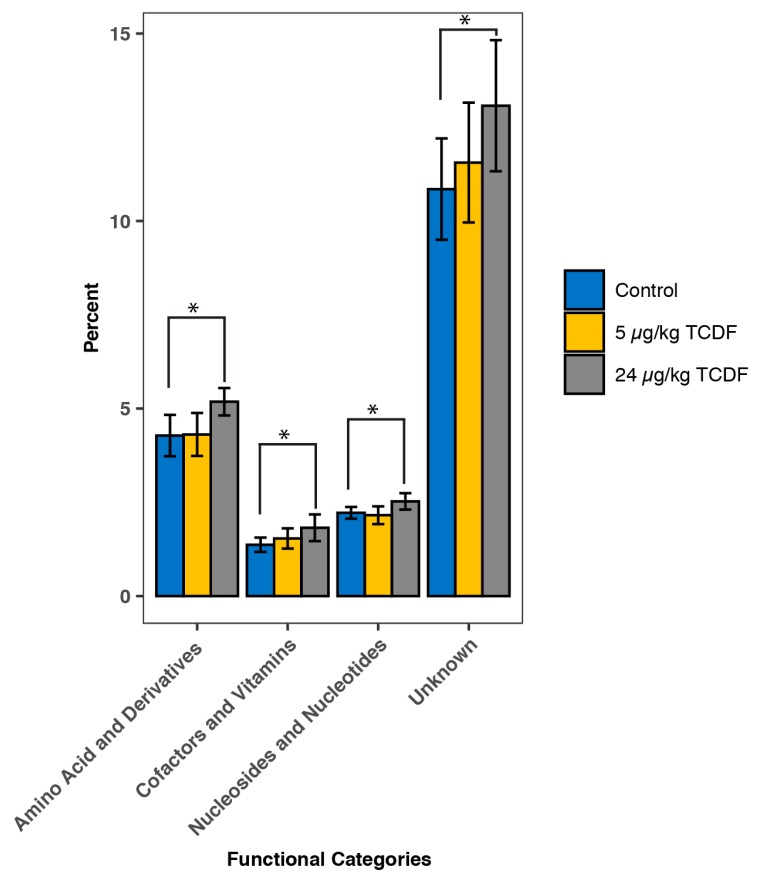
TCDF changes the functionality of the gut microbiome. A bar chart showing the changes in the major KEGG pathways after TCDF treatment using the SAMSA2 pipeline between the control (blue), and 5 (gold) and 24 µg/kg BW TCDF (grey). Data are presented as mean ± SD (*n* = 6 per group). * *p* < 0.05.

The pathways represented by KEGG categories from the SAMSA2 (Simple Annotation of Metatranscriptomes by Sequence Analysis) analysis are illustrated in Figure 3. Four significant KEGG categories increased after 24 µg/kg BW TCDF exposure, including amino acid metabolism, cofactors and vitamin metabolism, nucleoside and nucleotide metabolism, and pathways with an as of yet unknown function (Figure 3). There were no significant differences between 5 µg/kg BW TCDF and the control, as identified in Figure 3. Figure S2 illustrates the breakdown of the top 50 significant changes in gene expression. Of the top 50 significant changes in gene expression, 46 genes had increased expression after treatment with 24 µg/kg BW TCDF. Genes that decreased after 24 µg/kg BW TCDF exposure are putative preQ0 transporter (RNA metabolism), SOS-response repressor and protease LexA and Helicase loader DNAl (DNA metabolism), and sucrose operon repressor (carbohydrate metabolism). Of particular note, there were significant increases in the V-Type ATP synthase subunits B, E, K, and F genes and significant increases in the glutamate synthase gene, cysteine synthase gene, and threonine dehydratase gene (Figure S2).

Pathway enrichment was performed using the metatranscriptomics data. Table 4 reports all of the pathways that were significantly enriched and depleted. Of particular note, amino sugar and nucleotide sugar; amino acid biosynthesis; alanine, aspartate, and glutamate metabolism; starch and sucrose metabolism; valine, leucine, and isoleucine biosynthesis; and D-glutamine and D-glutamate metabolism were all significantly enriched after the 24 µg/kg BW TCDF treatment. LPS biosynthesis was significantly enriched after 24 µg/kg BW TCDF (*p* = 0.0355, Q = 0.195).

### 2.5. Modulation of Bacterial Pathways After TCDF Exposure

**Figure 4:**
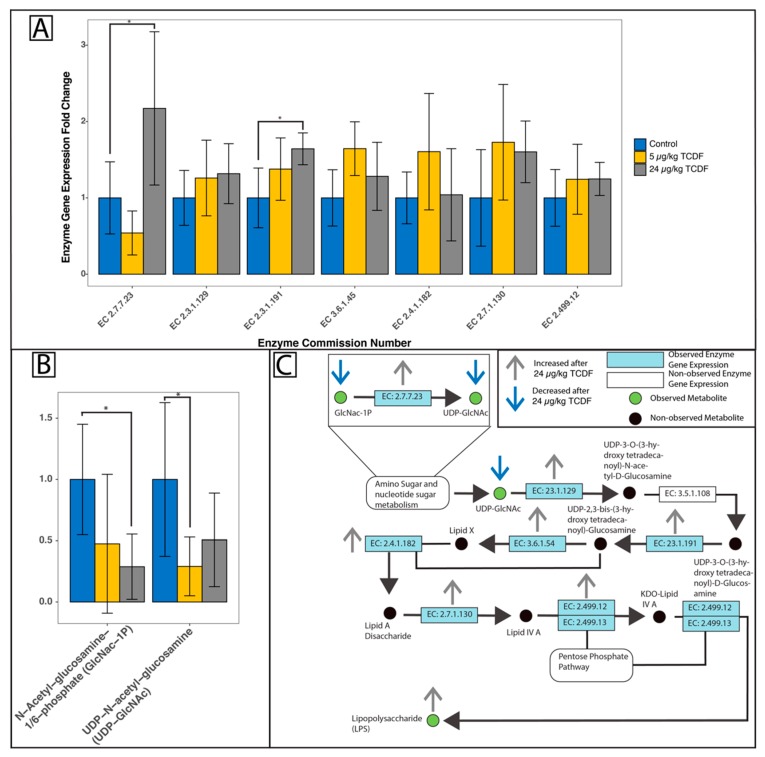
TCDF increases the expression of major enzymes within the LPS biosynthesis pathway. (A) All observed and relevant enzyme gene expression changes identified via metatranscriptomic analysis are reported as fold changes compared to the control average. (**B**) All observed and relevant metabolite changes after LC-MS analysis are reported as fold changes compared to the control average (**C**) Bacterial enzyme gene expression changes (**A**) and the observed metabolite changes (**B**) are mapped onto the LPS biosynthesis KEGG pathway. Green circles are observed metabolites; black circles are non-observed metabolites that are part of the pathway. Blue boxes are observed enzyme genes and white boxes are non-observed enzyme genes that are part of the LPS biosynthesis pathway. Grey arrows show enzyme genes or metabolites that are upregulated after exposure to 24 µg/kg BW TCDF, blue arrows show enzymes or metabolites that are downregulated after exposure to 24 µg/kg BW TCDF. Significant enzyme gene (**A**) or metabolite (**B**) changes after either 5 or 24 µg/kg BW TCDF exposure were discovered with LEfSe [25] or Student’s t test and are denoted with (*), *p* < 0.05. For full enzyme names, see Table S1.

The KEGG pathway for LPS biosynthesis shown in Figure 4C was annotated with the metatranscriptomic and metabolomic results. Two input metabolites for this pathway were identified, including UDP-N-acetyl-D-glucosamine and N-Acetyl-glucosamine-1/6-phosphate, which were significantly decreased after 5 and 24 µg/kg BW TCDF exposure, respectively (Figure 4B). The expression of UDP-3-O-[3-hydroxymyristoyl] glucosamine N-acyltransferase (EC 2.3.1.191) and N-acetylglucosamine-1-phosphate uridyltransferase (EC 2.7.7.23) were both significantly increased after 24 µg/kg BW TCDF exposure (Figure 4A). Additional gene expressions shown in Figure 4A were not significantly increased but were identified via metatranscriptomic analysis. All data displayed in Figure 4A and B were normalized to a control sample representing the mean and shows the fold change difference of each selected gene expression or metabolite. For complete enzyme gene (EC numbers to enzyme name) names, see Table S1. The significant increase in LPS biosynthesis after 24 µg/kg BW TCDF exposure could also be explained by the effects from TCDD and AHR activation on B cell development. It is understood that TCDD exposure can directly [29] and indirectly [30] (through AHR activation) delay the development of B cells. B cells have toll-like receptors, like TLR4, which are important for sensing LPS and responding with inflammatory cytokines. Since TCDD and AHR can impair the maturation process [31], the LPS increase could be compounded due to a lack of mature B cells that do not express TLR4 to sense LPS. During TCDD exposure, the host would have a dampened inflammatory response, due to the immature B cells. It was also noted that the major window for TCDD and AHR to affect B cells is 12 h after stimulation [29,32]. This is important because if there a sub-chronic exposure of TCDD, the B cells would be affected throughout the exposure, and when the exposure stopped there is a possibility of a buildup of LPS that was not cleared by the delayed B cells. An elevated level of LPS produced by the gut bacteria may cause an extended inflammatory response when normal B-cell activity is reached.

**Figure 5:**
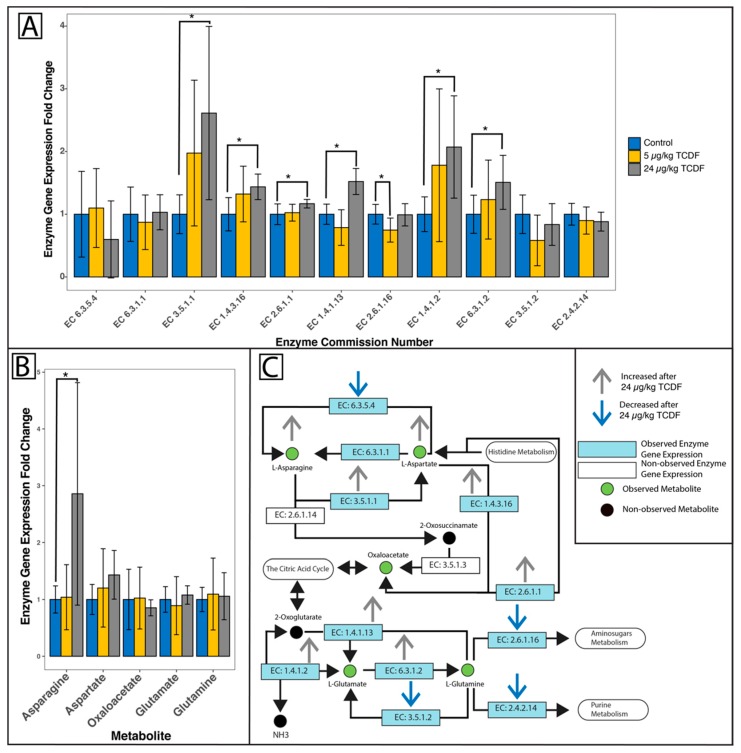
TCDF increases the utilization of glutamate seen in the alanine/aspartate/glutamate KEGG pathway. (**A**) All observed and relevant enzyme gene expression changes after metatranscriptomic analysis are reported as fold changes compared to the control mean. (**B**) All observed and relevant metabolite changes after LC-MS analysis are reported as fold changes compared to the control mean. (**C**) Together, the bacterial enzyme gene expression changes (**A**) and the observed metabolite changes (**B**) are mapped onto the alanine/aspartate/glutamate metabolism KEGG pathway. Green circles are observed metabolites; black circles are non-observed metabolites that are still part of the pathway. Blue boxes are observed enzyme genes and white boxes are non-observed enzyme genes that are still part of the alanine/aspartate/glutamate metabolism KEGG pathway. Grey arrows show enzyme genes or metabolites that are upregulated after exposure to 24 µg/kg BW TCDF, blue arrows show enzymes or metabolites that are downregulated after exposure to 24 µg/kg BW TCDF. Significant enzyme gene (**A**) or metabolite (**B**) changes after either 5 or 24 µg/kg BW TCDF exposure were discovered with LEfSe [25] or Student’s t tests and are denoted with (*), representing *p* < 0.05. For full enzyme names, see Supplementary Materials S1.

KEGG pathways for alanine, aspartate, and glutamate metabolism are illustrated in Figure 5C. Expression from 11 genes and 5 metabolites were detected with metatranscriptomics and metabolomics, respectively, with the expression of 6 genes significantly increased after 24 µg/kg BW TCDF and LEfSe analysis. The expression of the glutamate synthase gene (EC 1.4.1.13), glutamate dehydrogenase gene (EC 1.4.1.2), aspartate aminotransferase gene (EC 2.6.1.1), asparaginase gene (EC 3.5.1.1), glutamine synthetase gene (EC 6.3.1.2), and aspartate oxidase gene (EC 1.4.3.16) were all significantly increased after 24 µg/kg BW TCDF exposure (Figure 5A). Figure 5B shows the observed metabolites and a significant increase in asparagine after 24 µg/kg BW TCDF exposure. All gene expressions and metabolite levels were normalized to a control sample representing the mean and show fold change differences. For full EC names, see Table S1.

Glutamate catabolism has been shown to be an important indicator of bacterial stress [33]. Within the current study, there was a significant increase of glutamate synthase gene expression (EC 1.4.1.13) after TCDF treatment (Figure 5A). Glutamate synthase catalyzes the conversion of 2-oxo-glutarate (or glutamine) to glutamate. We found trends supporting this enzyme activity, including an increase (not significant) of glutamate and a decrease (not significant) of oxaloacetate after 24 µg/kg BW of TCDF treatment. There was a significant increase in asparagine (2.86 times higher) after 24 µg/kg BW exposure to TCDF (Table 1). Asparagine could be converted to aspartate by asparaginase (EC 3.5.1.1, significantly increased after 24 µg/kg BW exposure) and then be transformed into oxaloacetate by aspartate oxidase (EC 1.4.3.16, significantly increased after 24 µg/kg BW exposure) or aspartate aminotransferase (EC 2.6.1.1, significantly increased after 24 µg/kg BW exposure) and then enter the citric acid cycle (Figure 5C). There is a significant enrichment for the citric acid cycle after 24 µg/kg BW exposure to TCDF, which supports this assumption (Table 2). Asparagine is also directly used as an input material for cyanoamino acid metabolism and glycine, serine, and threonine metabolism and both pathways were seen to be significantly enriched after 24 µg/kg BW exposure to TCDF (Table 2). During the citric acid cycle, 2-oxoglutarate is produced, and 2-oxoglutarate is a reactant in the creation of glutamate through glutamate synthase (EC 1.4.12, significantly increased after 24 µg/kg BW exposure) and glutamine through glutamine synthase (EC 6.3.1.2, significantly increased after 24 µg/kg BW exposure) (Figure 5C).

The non-significant change seen with glutamate, coupled with the significant increase of glutamate synthase gene expression, could be due to increased downstream glutamate utilization. This was supported in Table 3, which showed that nitrogen metabolism was enriched after exposure to 24 µg/kg BW of TCDF. One major arm of nitrogen metabolism involves using the ammonia formed during the conversion of oxaloacetate to glutamate. The significant increase of the glutamate dehydrogenase expression (EC 1.4.1.2) to also support this claim (Figure 5C). Glutamate dehydrogenase has been reported to be an important regulator of ketoglutarate when bacterial cells are under stress from reactive oxygen species (ROS) [34]. The increase of glutamate utilization would most likely be in response to increased LPS production from the gut microbiome, which leaks out of the gut and activates toll-like receptor 4 (TLR4) and ultimately leads to increased host cytokine production and gut inflammation.

### 2.6. TCDF Exposure Causes an Increase in Circulating LPS

**Figure 6:**
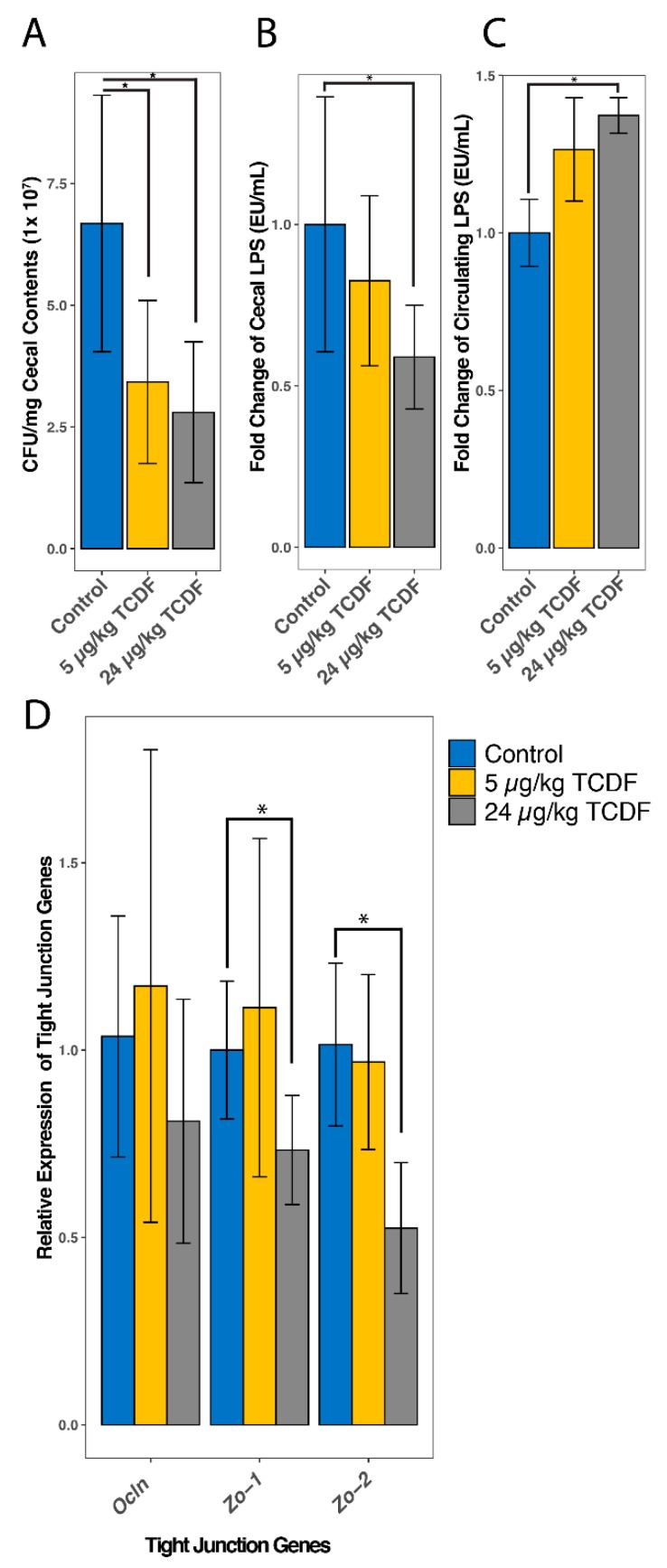
Circulating LPS increases and tight junction protein gene expression decreases after exposure to 24 µg/kg BW TCDF. (**A**) shows a bar chart of the average total bacteria (CFU) in one mg of cecal contents for the control, and 5 and 24 µg/kg BW TCDF. (**B**) is a bar chart showing the fold change of LPS present in the cecal contents between the control, and 5 and 24 µg/kg BW TCDF. Pairwise *T*-tests were done between the control and 5 µg/kg BW TCDF (*p* = 0.348), control and 24 µg/kg BW TCDF (*p* = 0.038), and 5 and 24 µg/kg BW TCDF (*p* = 0.243). (**C**) is a bar chart showing the fold change of circulating LPS in the serum between the control, and 5 and 24 µg/kg BW TCDF. Pairwise *T*-tests were done between the control and 5 µg/kg BW TCDF (*p* = 0.11), control and 24 µg/kg BW TCDF (*p* = 0.01), and 5 and 24 µg/kg BW TCDF (*p* = 0.12). (**D**) shows the fold change differences in the relative gene expression of occludin (*Ocln*), zonula occludens 1 (*Zo-1*), and zonula occludens 2 (*Zo-2*) after exposure to 5 and 24 µg/kg BW TCDF. After a student’s *T* test, the relative gene expression of both *Zo-1* (*p* = 0.018) and *Zo-2* (*p* = 0.004) were seen to significantly decrease after exposure to 24 µg/kg BW TCDF. Significance is denoted with (*) representing *p* < 0.05.

Circulating LPS was 1.27 times higher than the control following 5 µg/kg BW TCDF to 1.38 times higher than the control after 24 µg/kg BW TCDF (Figure 6C). Circulating LPS in mice treated with 24 µg/kg BW TCDF was significantly higher than the control after a pairwise t test with a Bonferroni correction to account for multiple comparisons (*p* = 0.031). An increase in LPS levels after TCDF exposure has been previously described [9], and additional increases in inflammatory factors like Tnf-α, IL-1β, and IL-10 have been reported [9,35,36,37]. In Figure 6B, cecal LPS was investigated and surprisingly there was a significant decrease in cecal LPS (1.61 times lower, *p* = 0.038) after 24 µg/kg BW TCDF as compared to the control. This discrepancy is likely due to the significant decrease (2.3 times lower, *p* = 0.01) in the total bacterial population seen in the cecal contents after treatment with 24 µg/kg BW TCDF (Figure 6A). LPS is primarily created by gram-negative bacteria, so the increase of LPS production after TCDF treatment is further supported by a significant increase in the gram-negative genus *Parasutterella* (Figure 2). Previous studies by our group and others noted that dietary TCDF (at 24 µg/kg body weight TCDF) and TCDD caused increases of inflammatory markers, like IL-1β, IL-10, TNF-α, LCN-2, and IgA [9,35,36,37]. Additionally, it has been reported that increased levels of circulating LPS can increase gut inflammation and contributes to obesity in rodents [19,38].

Figure 6D shows the changes in relative gene expression of occludin (*Ocln*), zonula occludens 1 (*Zo-1*), and zonula occludens 2 (*Zo-2*) in colon tissue. Both *Zo-1* (1.36 times lower, *p* = 0.018) and *Zo-2* (1.90 times lower, *p* = 0.0039) were significantly decreased after exposure to 24 µg/kg BW TCDF. Previous research supports the above claim by noting that an altered intestinal microbiome composition can reduce the expression of *Zo-1* and *Ocln*, both of which are responsible for creating tight junctions in the intestinal tissue [38]. Decreased gene expression of tight junction proteins can increase gut permeability, allowing for the release of LPS. Direct injections of LPS in Duroc X Landrace X Yorkshire piglets have been reported to decrease the expression of ZO-1 and occludin, increasing gut permeability [39]. Additionally, it has been reported that TCDD can cause increases in gut permeability, resulting in LPS escaping the gut into the serum [40]. The depleted gut bacterial population results in a lower amount of LPS present in the cecal contents, but the significant increase in the LPS biosynthesis pathway, and the observed decreases in tight junction proteins *Zo-1* and *Zo-2*, results in the significant increase of gut permeability and circulating LPS in the serum.

### 2.7. Overlapping Pathway Enrichment between Analytical Methods

**Figure 7:**
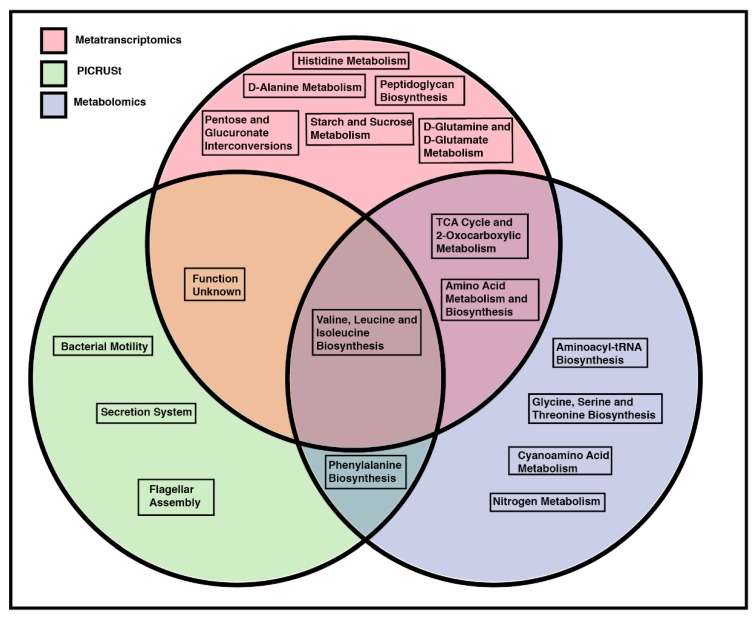
Metabolomics pathway enrichment may be a better predictor of the overall microbial bacteria function than PICRUSt. A Venn diagram of three functional prediction or pathway enrichment models was used to investigate the overlapping significantly enriched functions of the gut microbiome. Functional prediction with PICRUSt is represented by the green circle, pathway enrichment with metabolomics is represented by the lavender circle, and pathway enrichment with metatranscriptomic data is represented by the pink circle. Each pathway shown in Figure 7 was significant after LEfSe [25] (PICRUSt functional prediction) or after a student’s *T* test with an additional FDR correction, Q < 0.05 (both metabolomics and metatranscriptomics pathway enrichment). Pathways in circle overlaps are shared between the respective analytical techniques.

A Venn diagram (Figure 7) of significantly enriched or significantly upregulated pathways was generated based upon metatranscriptomic, metabolomic, and PICRUSt analysis and after 24 µg/kg BW TCDF treatment. Valine, leucine, and isoleucine biosynthesis were seen to be enriched in both metatranscriptomics and metabolomics, and also predicted to be upregulated after PICRUSt analysis after 24 µg/kg BW TCDF treatment. TCA cycle or 2-oxocarboxylic metabolism was enriched after both metabolomic and metatranscriptomic analysis, respectively, but not predicted to change after PICRUSt after 24 µg/kg BW TCDF treatment. Pathways with an unknown function were predicted to be upregulated by PICRUST and were significantly enriched after metatranscriptomic analysis but were not identified with metabolomic pathway enrichment. PICRUSt illustrates that amino sugar and nucleotide sugar metabolism and starch sucrose metabolism were both predicted to decrease after the 24 µg/kg BW TCDF treatment (Table 1). Amino sugar and nucleotide sugar metabolism and starch sucrose metabolism are both enriched after metatranscriptomic analysis(Figure 7, Table 4). Phenylalanine biosynthesis was predicted to be upregulated with PICRUSt (Table 1) and showed enrichment after metabolomic analysis. No significantly enriched or upregulated pathways were seen after the 5 µg/kg BW TCDF treatment.

Other than valine, leucine, and isoleucine biosynthesis and pathways with an unknown function, the PICRUSt results do not overlap with metatranscriptomics (Figure 7). Metatranscriptomics analysis reports the changes in gene expression in the intestinal microbiome and identified that amino acid metabolism and starch and sucrose metabolism (Table 4) are significantly enriched after 24 µg/kg BW TCDF exposure. PICRUSt predicts that amino acid metabolism and starch and sucrose metabolism would be significantly decreased after 24 µg/kg BW TCDF exposure (Table 1). This is likely due to a decrease in the functional capability of these two pathways after 24 µg/kg BW TCDF exposure but increases in gene expression (metatranscriptomics) from the bacterial species present after 24 µg/kg BW TCDF. Overlapping enriched pathways are reported between the metatranscriptomics and metabolomics data in Figure 7. There are some differences due to the host–microbiome metabolic axis, which involves crosstalk between the host and the microbiome via metabolites. Despite the metabolomic analysis on cecal contents, host metabolites will be present in the data, which can impact the microbial pathway enrichment based on the metabolomics data. Enrichment of the TCA cycle (Table 3) and enrichment of 2-oxocarboxylic metabolism (Table 4), overlapped in Figure 7. Additionally, enrichment of amino acid metabolism and biosynthesis (Table 4) and enrichment of glycine, serine, and threonine metabolism; phenylalanine, tyrosine, and tryptophan biosynthesis; cyanoamino acid metabolism; and valine, leucine, and isoleucine biosynthesis (Table 3) all overlapped in Figure 7. Pathway enrichment of the metatranscriptomic data and metabolite data show enrichment of amino acid utilization (Figure 7).

## 3. Conclusions

Elucidating the function of the intestinal microbiome remains a major milestone for drug discovery, risk assessment, and environmental toxicant research. We investigated the effects of 5 or 24 µg/kg BW exposures to TCDF on the mouse gut microbiome. Despite the limited taxonomic changes in the intestinal microbiome, there were large shifts in intestinal microbiome functionality after 24 µg/kg BW exposures to TCDF. Through metatranscriptomics, increases of LPS biosynthesis and the alanine/aspartate/glutamate metabolism pathways were identified. Data from metabolomics and LAL assays were integrated with the metatranscriptomic results and supported an increase of LPS biosynthesis. LPS was significantly decreased in cecal contents, likely due to a depleted gut bacterial population and an increase of gut permeability as LPS was significantly increased in the serum after 24 µg/kg BW exposures to TCDF due to increased LPS biosynthesis, coupled with the increase of gut permeability. These results support a potential relationship where strong AHR agonists like TCDF can induce LPS biosynthesis in the intestinal microbiome while simultaneously increasing gut permeability and result in an increase of circulating LPS in the serum. Overall, additional investigation into the in vivo relationship between bacterial-derived LPS, sustained activation of AHR by TCDD, and host immunity is warranted.

## 4. Materials and Methods

### 4.1. Animals and Diets

Mouse experimental procedures were performed according to the National Institutes of Health (NIH) guidelines and approved by the Pennsylvania State University Institutional Animal Care and Use Committee (The IACUC approval number is 47231. Approval was granted 3/19/2017 and will expire 4/20/2020). Four-week-old male C57BL/6J mice (22–25 g) were purchased from the Jackson Laboratory and housed at the Centralized Biological Laboratory of Pennsylvania State University (University Park, PA). Mice (*n* = 18) were acclimatized for one week and then underwent one week of pill training. Mice were then fed untreated dough pills (*n* = 6) or dough pills containing 5 µg/kg BW (*n* = 6) or 24 µg/kg BW (*n* = 6) TCDF for five consecutive days (one pill per mouse per day). Blood, liver, and cecal contents were collected immediately following euthanasia by CO_2_ asphyxiation. All samples were stored at −80 °C until analysis.

### 4.2. 16S rRNA Gene Amplicon Sequencing and Analysis

Cecal contents were extracted from the mice and bacterial DNA was isolated with the E.Z.N.A stool pathogen kit from OMEGA according to the manufacturer’s specifications. Bacterial DNA levels were assayed via Nanodrop (ThermoFisher). Aliquots were taken from the bacterial DNA extracts and all samples were brought to a concentration 10 ng/µL. Using 1 µL of each aliquoted sample and the Platinum SuperFi master mix (Invitrogen), amplification of the V4 (515F and 806R) region was completed with PCR (1 cycle at 98 °C (2 min); 25 cycles of 98 °C (10 s), 56.6 °C (20 s), 72 °C (15 s); and 1 cycle at 72 °C (5 min)). Amplicons were then checked on a 1X agarose gel and then submitted to the Pennsylvania State University Genomics Core Facility for 250 × 250 paired end Illumina Miseq sequencing. Blank samples and mock community standards (ZymoBIOMICS live microbial community standard) were analyzed with the test samples to check for contamination and check the error rate for the extraction and sequencing method, respectively. Raw data was returned and 16S rRNA gene amplicon analysis was completed using mothur software (version 1.39.5) and the SILVA database (2017 version) [41,42]. After mothur, the OTUs (operational taxonomic units) were mapped onto a user-created phylogenic tree (made in mothur) to complete variance-adjusted weighted Unifrac. Variance-adjusted weighted unifrac investigates the phylogenic distances of all the abundant species in a microenvironment and randomizes the leaves of the phylogenic tree to allow for a normal distribution of the variances and increases the power of the metric above both regular weighted unifrac and unweighted unifrac analysis [43].

The raw 16S data were realigned with the GreenGenes (gg_13_5_99) database for a PICRUSt analysis [44]. PICRUSt uses the taxonomically assigned 16S data to predict the function of the sample microbiome. PICRUSt significance was determined with LEfSe (linear discriminant analysis effect size) analysis, which utilizes a Kruskal–Wallis and a Wilcoxon rank-sum test to report predicted functional changes that are significantly different and biologically relevant [25].

### 4.3. LC-MS-Based Metabolomics

Cecal sample preparation for LC-MS and data analysis was performed as previously described [45]. In brief, cecal samples were extracted twice with 1 mL of cold 50% methanol containing 1 µM chlorpropamide. After homogenization, the samples were freeze-thawed three times, followed by centrifugation at 15,000× *g* for 10 min. The combined supernatants were dried in a vacuum and reconstituted in 60 μL of 3% methanol. After centrifugation (15,000× *g* for 2 min), 50 μL of supernatants were transferred to autosampler vials for LC-MS analysis. Metabolites were measured on a Dionex Ultimate 3000 quaternary HPLC system (Thermo Fisher Scientific, Waltham, MA) coupled to an Exactive^TM^ Plus Orbitrap mass spectrometer (Thermo Fisher Scientific) with a Hydro-RP C18 column (2.1 × 100 mm × 2.5 µm particle size; Phenomenex, Torrance, CA, USA). LC-MS data were analyzed by an open-source software pipeline MS-DIAL [46] for compound discovery and MetaboAnalyst (web version 4) [26,27,28] for pathway enrichment. Significance was determined with a student’s *T* test, *p* < 0.05 and a false discovery rate Q < 0.05.

### 4.4. Metatranscriptomic Analysis

In total, 100 mg of cecal contents were used for RNA isolation using a 1:10 trizol/chloroform extraction. Total RNA was then measured and checked via an Agilent Bioanalyzer. The RiboZero isolation kit from Illumina was used to remove 16S and 23S rRNA from the samples. The remaining mRNA was checked again with the Agilent Bioanalyzer to ensure depletion of the 16S and 23S fractions. All mRNA was then submitted to the Pennsylvania State University Genomic Sequencing core for single-end RNAseq analysis on the Illumina Hiseq (150 bp). Unfiltered raw data was returned and analysis was completed with the SAMSA2 (version 2.0.0) (Simple Annotation of Metatranscriptomes by Sequence Analysis) pipeline [47]. SAMSA2 [47] uses PEAR (version 0.9.8) [48], Trimmomatic (version 0.36) [49], and ShortMeRNA(version 2.1) [50] to merge the paired reads (PEAR) and trim low quality reads (Trimmomatic) and rRNA (ShortMeRNA) from the pairs. Then, SAMASA2 utilizes DIAMOND (version 0.9.8) [51] to annotate the paired mRNA reads to the RefSeq database [52]. After annotation, SAMASA2 incorporates many R (version 3.5.2) packages like DESeq2 [53] to further analyze the mRNA data and produces gene expression data for KEGG enzymes (classified with enzyme commission [EC] numbers) that will map onto annotated KEGG pathways. Enzyme gene expression data was first normalized to the total read count for each sample (to provide percentiled data), then used for downstream analysis with LEfSe [25]. The significantly changed gene expression pathways were then mapped to annotated KEGG pathways. Additionally, the gene expression data was analyzed with MetaboAnalyst [26,27,28] for pathway enrichment. Significance was determined with a student’s *T* test, *p* < 0.05 and a false discovery rate Q < 0.05.

### 4.5. Lipopolysaccharide Detection

The Pierce^TM^ LAL Chromogenic Endotoxin Quantitation Kit (Thermo Scientific) was used according to the manufacturer’s protocol. A standard curve was created with the provided endotoxin, diluted to 1, 0.5, 0.25, and 0.1 EU/mL. Serum samples were run in duplicate (*n* = 3 for control, 5 µg/kg BW TCDF, and 24 µg/kg BW TCDF). Plates were analyzed for absorbance at 405 nm on a Synergy HTX multimode plate reader (BioTek).

Then, 10 mg of cecal contents were first diluted in 10 mL of endotoxin-free water. A 500-µL aliquot was removed and sonicated with a probe sonicator. In total, 6 µL of each sonicated aliquot was then diluted in 1 mL of endotoxin-free water. The diluted sample was then used for the Pierce^TM^ LAL Chromogenic Endotoxin Quantitation assay according to the manufacturer’s protocol.

### 4.6. Total Bacteria Quantification

An aliquot of extracted bacterial DNA was diluted to 1 ng/µL for each sample. Then, 3 µL of DEPC water, 1 µL of universal forward primer (AGAGTTTGATCCTGGCTCAG), 1 µL of universal reverse primer (CTGCTGCCTCCCGTAGGAGT), 6 µL of SYBR green QPCR master mix (BioRad), and 1 µL of each sample (*n* = 18) were added to a 96-well plate for QPCR analysis. QPCR was ran with the following conditions: 95 °C for 20 s; 95 °C for 0.01 s; 60 °C for 20 s; 95 °C for 15 s; 60 °C for 15 s; and 95 °C for 15 s, for 45 cycles. ΔΔCT values were obtained and a previously developed standard curve with the line of best fit equation (1) was used to determine CFU counts of the samples.

y = (6 × 10^12^) (e^−0.685x^) (1)

where y = CFU and x = ΔΔCT values.

CFU levels were then divided by the concentration of the original bacterial DNA extract sample and also divided by the weight of the cecal contents used for bacterial DNA extraction, resulting in the CFU per mg of cecal contents.

### 4.7. Quantitative Real-Time Polymerase Chain Reaction (qPCR) Analysis of Tight Junction Proteins

RNA was extracted from ~35 mg of colon tissue with TRIzol reagent (Invitrogen). RNA extracts were normalized to 500 ng/µL, and 2 µL of RNA was used to synthesize cDNA with qScript cDNA SuperMix (Quanta bio). Then, 1 µL of cDNA was mixed with Fast SYBR Green Master Mix (applied Biosystems) and appropriate primers for occludin (*Ocln*), zonula occludens 1 (*Zo-1*), and zonula occludens 2 (*Zo-2*) (primer sequences are available in Table S4) for qPCR analysis (40 cycles of 95 °C for 20 s, 95 °C for 0.01 s, 60 °C for 20 s, 95 °C for 15 s, 60 °C for 16 s, and 95 °C for 15 s) on the ABI Prism 7900 HT Fast Real-Time PCR sequence detection system (Applied Biosystems). All qPCR results were normalized to *Gapdh* and analyzed with the ΔΔCT method.

## Figures and Tables

**Table 1:** Predicted functional changes by PICRUSt. Significant predicted pathway changes after PICRUSt analysis. LEfSe (Linear Discriminat Analysis Effect Size) [25] was used to determine the biological relevance and statistical significance with a combination of a Kruskal–Wallis sum rank test and a Wilcoxon rank sum test, respectively.

Pathway	*p* Value	Upregulated (↑) or Downregulated (↓)
Arginine and proline metabolism	0.01	↓
Amino sugar and nucleotide sugar metabolism	0.02	↓
Otherion coupled transporters	0.02	↓
Lysine biosynthesis	0.02	↓
Glyoxylate and dicarboxylate metabolism	0.02	↓
Starch and sucrose metabolism	0.02	↓
Cysteine and methionine metabolism	0.02	↓
Valine leucine and isoleucine degradation	0.02	↑
Secretion system	0.02	↑
Bacterial motility proteins	0.02	↑
Flagellar assembly	0.02	↑
Function unknown	0.04	↑
Phenylalanine metabolism	0.004	↑

**Table 2:** Significant changes in metabolites after TCDF exposure.

Metabolite Name	Control	5 µg/kg TCDF	24 µg/kg TCDF
3-Phosphoglycerate	7.92 ± 6.00	3.31 ± 3.07	1.86 ± 0.77 *
6-Phospho-D-gluconate	0.29 ± 0.27	0.11 ± 0.14	0.03 ± 0.02 *
Aconitate	2.83 ± 1.23	1.82 ± 1.03	1.57 ± 0.47 *
Anthranilate	0.38 ± 0.09	0.37 ± 0.12	0.30 ± 0.04 *
Asparagine	2.46 ± 0.59	2.56 ± 1.41	7.03 ± 4.81 *
CDP	0.16 ± 0.06	0.09 ± 0.09	0.07 ± 0.04 *
Citrulline	133.18 ± 31.27	67.80 ± 60.84 *	165.57 ± 53.93
dGDP	1.42 ± 0.52	1.22 ± 1.22	0.70 ± 0.37 *
Dihydroxy-acetone-phosphate	3.81 ± 2.69	2.52 ± 2.83	0.95 ± 0.60 *
FMN	3.38 ± 0.94	1.74 ± 1.26 *	1.41 ± 0.68 *
Fumarate	7.47 ± 2.42	13.08 ± 7.26	15.21 ± 4.50 *
GDP	0.36 ± 0.10	0.28 ± 0.23	0.18 ± 0.08 *
Gluconolactone	10.20 ± 7.46	3.20 ± 1.68 *	5.64 ± 5.66
Glycine	15.34 ± 3.61	17.72 ± 10.54	21.71 ± 4.50 *
Hydroxyproline/Aminolevulinate	3.42 ± 0.84	2.31 ± 1.39	1.30 ± 0.35 *
IMP	0.77 ± 0.37	0.38 ± 0.29	0.30 ± 0.26 *
Leucine/Isoleucine	331.04 ± 99.91	447.25 ± 261.53	599.00 ± 134.45 *
N-Acetyl-glucosamine-1/6-phosphate	26.03 ± 11.66	12.33 ± 14.70	4.36 ± 4.34 *
Phosphoenolpyruvate	0.89 ± 0.71	0.36 ± 0.31	0.17 ± 0.09 *
Prephenate	1.33 ± 0.62	1.22 ± 0.52	0.73 ± 0.17 *
Proline	56.59 ± 21.32	63.79 ± 44.10	83.57 ± 20.20 *
Pyridoxamine	0.26 ± 0.06	0.24 ± 0.10	0.18 ± 0.06 *
Pyruvate	8.32 ± 4.57	3.84 ± 1.97 *	6.02 ± 3.53
Serine	33.77 ± 7.94	42.06 ± 25.03	53.34 ± 13.06 *
Glycerol-3-phosphate	64.65 ± 30.11	55.85 ± 72.70	25.35 ± 16.89 *
Threonine/Homoserine	60.19 ± 12.41	59.96 ± 32.22	85.89 ± 11.08 *
Tryptophan	105.14 ± 26.96	128.27 ± 73.36	167.80 ± 39.14 *
UDP	0.24 ± 0.08	0.17 ± 0.16	0.12 ± 0.08 *
UDP-D-glucose	0.15 ± 0.08	0.07 ± 0.05 *	0.06 ± 0.06 *
UDP-N-acetyl-glucosamine	0.12 ± 0.07	0.04 ± 0.03 *	0.05 ± 0.05
Xanthine	30.00 ± 8.01	14.65 ± 9.71 *	21.14 ± 9.89
Xanthosine	55.04 ± 12.35	30.42 ± 24.52 *	26.54 ± 15.38 *
Xanthosine-5-phosphate	0.73 ± 0.40	0.36 ± 0.31	0.27 ± 0.22 *

Significant metabolite changes identified after 5 and 24 µg/kg BW TCDF exposure. All data shown are mean ± SD. * represent significance according to a Student’s t test (*p* < 0.05).

**Table 3:** Pathway enrichment analysis of metabolomic data.

Pathway	*P* Value	*Q* Value	Enriched (↑) or Depleted (↓)
Aminoacyl-tRNA biosynthesis *	2.06 × 10^−6^	1.64 × 10^−4^	↑
Glycine, serine and threonine metabolism *	1.89 × 10^−5^	7.55 × 10^−4^	↑
Phenylalanine, tyrosine, and tryptophan biosynthesis *	2.82 × 10^−4^	0.007	↑
Cyanoamino acid metabolism *	8.77 × 10^−4^	0.02	↑
Nitrogen metabolism *	0.001	0.02	↑
Citrate cycle (TCA cycle) *	0.002	0.02	↑
Valine, leucine and isoleucine biosynthesis *	0.004	0.05	↑
Glycerolipid metabolism	0.007	0.07	↓
Sulfur metabolism	0.02	0.18	↑
Purine metabolism	0.02	0.20	↓
Alanine, aspartate, and glutamate metabolism	0.03	0.25	↑
Pyrimidine metabolism	0.04	0.25	↓

Pathway enrichment analysis using the metabolite data comparing control mice with 24 µg/kg BW TCDF exposure mice. All pathways shown have a significant *p* value (*p* < 0.05) after Student’s t test. * represent enriched pathways that have a significant Q value (Q < 0.05) after false discovery rate (FDR) correction.

**Table 4:** Pathway enrichment analysis of metatranscriptomic data.

Pathway	*P* Value	*Q* Value	Enriched (↑) or Depleted (↓)
Biosynthesis of amino acids	1.24 × 10^−17^	1.84 × 10^−15^	↑
Amino sugar and nucleotide sugar metabolism	1.01 × 10^−5^	7.47 × 10^−4^	↑
One carbon pool by folate	7.05 × 10^−5^	3.48 × 10^−4^	↑
Histidine metabolism	3.5 × 10^−4^	0.01	↑
Polyketide sugar unit biosynthesis	5.58 × 10^−8^	0.01	↓
Alanine, aspartate and glutamate metabolism	6.05 × 10^−4^	0.0124	↑
Pantothenate and CoA biosynthesis	6.49 × 10^−4^	0.01	↓
Pentose and glucuronate interconversions	6.72 × 10^−4^	0.01	↑
Pyruvate metabolism	0.001	0.02	↓
Terpenoid backbone biosynthesis	0.001	0.02	↑
Carbon fixation in photosynthetic organisms	0.001	0.02	↓
Peptidoglycan biosynthesis	0.001	0.02	↑
Starch and sucrose metabolism	0.003	0.03	↑
D-Alanine metabolism	0.004	0.04	↑
2-Oxocarboxylic acid metabolism	0.004	0.04	↑
Valine, leucine and isoleucine biosynthesis	0.004	0.04	↑
Vitamin B6 metabolism	0.005	0.04	↑
Streptomycin biosynthesis	0.005	0.04	↑
D-Glutamine and D-glutamate metabolism	0.006	0.05	↑
Drug metabolism—other enzymes	0.006	0.05	↑
Carbon fixation pathways in prokaryotes	0.007	0.05	↑
Propanoate metabolism	0.02	0.103	↓
Carbapenem biosynthesis	0.02	0.15	↓
Novobiocin biosynthesis	0.02	0.15	↑
Glycolysis/Gluconeogenesis	0.03	0.18	↓
Lipopolysaccharide biosynthesis	0.03	0.19	↑
Cysteine and methionine metabolism	0.04	0.20	↑
Nicotinate and nicotinamide metabolism	0.04	0.20	↑
Arginine and proline metabolism	0.04	0.20	↑
Lysine biosynthesis	0.04	0.20	↑

Pathway enrichment analysis using the metatranscriptomic results comparing control mice with 24 µg/kg BW TCDF exposure mice. All pathways shown have a significant *p* value (*p* < 0.05) after a student’s t test. * represent enriched pathways that have a significant Q value (Q < 0.05) after false discovery rate (FDR) correction.

## Supplementary Materials

The following are available online at https://www.mdpi.com/2218-1989/10/1/1/s1, Figure S1: Bacteroidetes to Firmicutes ratio after exposure to TCDF; Figure S2: Top 50 significant KEGG enzymes and COGs after 24 µg/kg BW TCDF exposure TCDF exposure; Table S1: Enzyme names for EC numbers; Table S2: Normalized Metabolite Table for Identified Metabolites; Table S3: Normalized Metabolite Table for Unidentified Metabolites; Table S4: Primer Sequences for qPCR Analysis of Tight Junction Proteins.
