# Supplementary material for: Metatranscriptomic Analysis of the Mouse Gut Microbiome Response to the Persistent Organic Pollutant 2,3,7,8-Tetrachlorodibenzofuran
Source: Metabolites. 2019 Dec 18;10(1):1. doi: 10.3390/metabo10010001 (PMC7022680; doi:10.3390/metabo10010001)

Supplemental Table S1: Enzyme names for EC numbers

A table showing the respective Enzyme name for each EC number used in this manuscript.

EC NUMBERS	ENZYME NAMES
**EC 1.4.1.13**	Glutamate synthase [NADPH] small chain
**EC 1.4.1.2**	NAD-specific glutamate dehydrogenase
**EC 2.6.1.1**	Aspartate aminotransferase
**EC 3.5.1.1**	L-asparaginase
**EC 6.3.1.2**	Glutamine synthetase type I
**EC 3.5.1.2**	Glutaminase
**EC 1.4.1.4**	NADP-specific glutamate dehydrogenase
**EC 2.6.1.16**	Glucosamine--fructose-6-phosphate aminotransferase [isomerizing]
**EC 2.4.2.14**	Amidophosphoribosyltransferase
**EC 1.4.3.16**	L-aspartate oxidase
**EC 6.3.1.1**	Aspartate ammonia ligase
**EC 6.3.5.4**	Asparagine synthetase [glutamine-hydrolyzing]
**EC 2.1.3.2**	Aspartate carbamoyltransferase
**EC 23.1.191**	UDP-3-O-[3-hydroxymyristoyl] glucosamine N-acyltransferase
**EC 2.5.1.55**	2-Keto-3-deoxy-D-manno-octulosonate-8-phosphate synthase
**EC 5.3.1.13**	Arabinose 5-phosphate isomerase
**EC 2.7.1.130**	Tetraacyldisaccharide 4'-kinase
**EC 23.1.129**	Acyl-[acyl-carrier-protein]--UDP-N-acetylglucosamine O-acyltransferase
**EC 2.499.12**	3-deoxy-D-manno-octulosonic-acid transferase
**EC 3.6.1.54**	UDP-2,3-diacylglucosamine hydrolase
**EC 2.4.1.182**	Lipid-A-disaccharide synthase
**EC 2.7.7.23**	N-acetylglucosamine-1-phosphate uridyltransferase

Supplemental Table S2: Normalized Metabolite Table for Identified Metabolites

A list of all the M/Z and retention time values for all identified metabolites after LC-MS analysis.

Metabolite name	Average Rt(min)	Average Mz	Average Control	Average 5 µg/kg BW TCDF	Average 24 µg/kg BW TCDF
**2-Aminooctanoic acid**	11.82	158.119	5.296 ± 1.682	13.355 ± 18.779	5.464 ± 1.308
**2-Isopropylmalic acid**	14.1	175.061	22.902 ± 9.518	14.191 ± 11.670	15.602 ± 9.596
**2-Keto-D-gluconate**	5.03	193.035	115.752 ± 93.995	58.629 ± 55.642	56.950 ± 36.132
**2-Keto-isovalerate**	13.28	115.04	3.857 ± 5.419	1.439 ± 0.569	2.060 ± 1.728
**2,3-Dihydroxybenzoic acid**	13.68	153.02	0.717 ± 0.225	0.729 ± 0.335	0.567 ± 0.171
**3-Hydroxybutyric acid**	3.83	103.04	0.447 ± 0.068	0.526 ± 0.078	0.494 ± 0.142
**3-Methylphenylacetic acid**	15.38	149.061	0.685 ± 0.210	1.192 ± 1.193	0.698 ± 0.125
**3-Phospho-serine**	8.55	184.002	0.847 ± 0.687	0.397 ± 0.330	0.372 ± 0.135
**3-Phosphoglycerate**	13.54	184.986	7.918 ± 6.002	3.306 ± 3.068	1.855 ± 0.774

**3-S-Methylthiopropionate**	12.26	119.017	3.279 ± 7.484	0.117 ± 0.272	0.729 ± 1.337
**4-Aminobutyrate**	3.76	102.056	23.115 ± 6.021	20.191 ± 11.987	25.644 ± 4.375
**4-Hydroxyphenyllactate**	11.23	181.051	0.506 ± 0.163	0.377 ± 0.131	0.329 ± 0.167
**4-Phosphopantothenate**	13.92	298.07	0.158 ± 0.149	0.079 ± 0.097	0.045 ± 0.031
**4-Pyridoxic acid**	14.17	182.046	93.972 ± 19.820	74.319 ± 40.942	75.610 ± 23.435
**5-Methoxytryptophan**	8.54	233.093	0.646 ± 0.187	0.594 ± 0.342	0.597 ± 0.129
**6-Phospho-D-gluconate**	13.35	275.018	0.289 ± 0.272	0.110 ± 0.141	0.028 ± 0.018
**Acetoacetate**	8.02	101.024	1.882 ± 0.621	2.035 ± 0.587	1.507 ± 0.343
**Acetyl-aspartate**	13.07	174.041	321.136 ± 132.666	176.155 ± 114.266	212.430 ± 79.121
**Acetyl-glycine**	7.6	116.035	39.345 ± 10.914	31.572 ± 22.901	42.923 ± 7.448
**Acetyllysine**	1.29	187.109	1.864 ± 0.696	1.365 ± 0.807	1.709 ± 0.673
**Aconitate**	13.98	173.009	2.831 ± 1.225	1.823 ± 1.034	1.575 ± 0.465
**Adenosine**	1.26	266.088	0.797 ± 0.220	0.612 ± 0.385	0.877 ± 0.441

**Alanine/Sacrosine**	1.21	88.04	45.047 ± 10.018	47.198 ± 25.202	56.028 ± 9.824
**Alpha-ketoglutarate**	13.27	145.014	7.935 ± 5.502	3.667 ± 1.616	6.098 ± 3.352
**Aminoadipic acid**	3.52	160.062	3.703 ± 0.501	3.444 ± 1.837	3.399 ± 0.980
**AMP**	11.34	346.056	1.788 ± 2.188	4.689 ± 6.733	1.577 ± 1.365
**Anthranilate**	13.44	136.04	0.384 ± 0.088	0.373 ± 0.124	0.297 ± 0.038
**Ascorbic acid**	6.67	175.025	0.287 ± 0.043	0.207 ± 0.094	0.269 ± 0.138
**Asp-Leu-His (DLH)**	3.9	382.173	0.453 ± 0.231	0.398 ± 0.254	0.557 ± 0.265
**Asp-Leu/Ile-Ser (DLS)**	11.55	332.147	0.713 ± 0.201	0.640 ± 0.356	0.742 ± 0.356
**Asparagine**	1.19	131.046	2.460 ± 0.588	2.557 ± 1.408	7.028 ± 4.813
**Aspartate**	4.14	132.03	326.767 ± 86.610	392.581 ± 224.693	467.957 ± 139.292
**ATP**	15.34	505.989	0.014 ± 0.017	0.021 ± 0.021	0.007 ± 0.009
**CDP**	13.43	402.012	0.157 ± 0.060	0.086 ± 0.086	0.073 ± 0.040
**Chlorpropamide**	17.31	275.027	34.560 ± 14.897	30.449 ± 17.166	32.712 ± 6.858

**Citraconic acid**	13.36	129.02	0.801 ± 0.197	0.942 ± 0.256	0.654 ± 0.197
**Citrate/isocitrate**	13.43	191.02	3.388 ± 0.965	2.962 ± 1.610	3.096 ± 1.449
**Citrulline**	1.28	174.088	133.177 ± 31.274	67.795 ± 60.845	165.568 ± 53.929
**CMP**	8.63	322.045	2.191 ± 0.976	1.940 ± 1.258	2.337 ± 1.234
**CTP**	15.07	481.978	0.000 ± 0.000	0.001 ± 0.003	0.000 ± 0.000
**Cystathionine**	1.25	221.06	1.928 ± 0.635	1.989 ± 1.314	2.874 ± 1.541
**Cytosine**	1.14	110.036	0.150 ± 0.042	0.135 ± 0.066	0.118 ± 0.059
**D-Sedoheptulose-1/7-phosphate**	7.34	289.033	47.904 ± 56.461	23.269 ± 32.253	3.049 ± 2.178
**dAMP**	11.91	330.061	4.018 ± 4.731	14.481 ± 20.749	3.780 ± 5.176
**dCMP**	9.73	306.05	6.825 ± 6.288	20.055 ± 27.108	7.396 ± 9.132
**Deoxyinosine**	3.92	251.077	0.098 ± 0.120	0.016 ± 0.012	0.041 ± 0.041
**Deoxyribose-phosphate**	7.83	213.017	6.653 ± 6.693	17.120 ± 26.967	6.057 ± 6.843
**dGDP**	14.03	426.023	1.421 ± 0.515	1.223 ± 1.219	0.700 ± 0.367

**Dihydroorotate**	7.2	157.026	1.972 ± 1.180	2.416 ± 3.589	3.095 ± 2.980
**Dihydroxy-acetone-phosphate**	9.34	168.991	3.810 ± 2.687	2.523 ± 2.831	0.955 ± 0.599
**dTMP**	11.48	321.05	18.883 ± 26.560	50.190 ± 84.280	13.184 ± 13.072
**Erythrose-4-phosphate**	7.14	199.002	0.442 ± 0.528	0.180 ± 0.241	0.037 ± 0.028
**FMN**	14.47	455.098	3.376 ± 0.941	1.736 ± 1.258	1.411 ± 0.682
**Folate**	14	440.133	0.364 ± 0.213	0.237 ± 0.158	0.209 ± 0.083
**Fructose-1,6-bisphosphate**	13.64	338.989	0.181 ± 0.157	0.046 ± 0.064	0.042 ± 0.047
**Fructose-6-phosphate**	8.07	259.023	4.912 ± 4.851	2.295 ± 2.640	1.020 ± 0.603
**Fumarate**	13.49	115.004	7.472 ± 2.418	13.075 ± 7.257	15.210 ± 4.500
**GDP**	13.72	442.018	0.363 ± 0.098	0.284 ± 0.230	0.177 ± 0.084
**Glucarate**	13.18	209.03	3.513 ± 2.313	2.726 ± 2.023	2.424 ± 0.735
**Gluconate**	5.06	195.051	16.803 ± 5.084	9.972 ± 6.002	10.334 ± 6.202
**Glucono-_-lactone**	7.01	177.041	10.201 ± 7.461	3.196 ± 1.677	5.644 ± 5.659

**Glucose**	1.27	179.056	267.777 ± 140.902	197.627 ± 200.602	250.676 ± 169.947
**Glucose-1/6-phosphate**	7.14	259.023	106.199 ± 130.633	46.810 ± 65.739	6.967 ± 5.981
**Glutamate**	3.78	146.046	3146.460 ± 710.167	2801.547 ± 1606.715	3394.040 ± 508.440
**Glutamine**	1.24	145.062	53.953 ± 11.545	59.000 ± 34.021	57.032 ± 22.312
**Glutathione**	7.89	306.077	0.246 ± 0.159	0.570 ± 0.617	0.232 ± 0.141
**Glycine**	1.19	74.024	15.340 ± 3.615	17.724 ± 10.538	21.709 ± 4.503
**GMP**	10.8	362.051	1.164 ± 0.551	0.948 ± 0.754	0.915 ± 0.516
**GTP**	15.17	521.984	0.000 ± 0.000	0.001 ± 0.003	0.000 ± 0.000
**Guanine**	3.47	150.042	0.437 ± 0.142	0.492 ± 0.284	0.481 ± 0.158
**Histidine**	1.13	154.062	3.653 ± 1.548	4.075 ± 2.385	4.625 ± 1.339
**Hydroxyisocaproic acid**	14.4	131.071	21.704 ± 13.429	19.808 ± 18.938	14.765 ± 10.673
**Hydroxyphenylacetic acid**	14.61	151.04	0.546 ± 0.116	0.529 ± 0.082	0.447 ± 0.081
**Hydroxyproline/Aminolevulinate**	1.23	130.051	3.416 ± 0.836	2.314 ± 1.386	1.305 ± 0.347

**Ile-Asp-Ser (IDS)**	8.22	332.147	0.845 ± 0.339	0.514 ± 0.291	0.686 ± 0.227
**IMP**	10.78	347.04	0.771 ± 0.375	0.378 ± 0.289	0.299 ± 0.255
**Indole-3-carboxylic acid**	14.27	160.041	1.273 ± 0.342	1.113 ± 0.592	1.216 ± 0.238
**Inosine**	3.69	267.072	1.855 ± 0.477	1.373 ± 1.498	1.150 ± 0.951
**Ketoleucine**	14.27	129.056	1.097 ± 0.242	1.931 ± 1.846	1.019 ± 0.165
**Lactate**	7.21	89.024	416.595 ± 202.422	391.389 ± 273.981	368.120 ± 147.177
**Leu-Asp (LD)**	8.51	245.114	1.135 ± 0.431	1.093 ± 0.582	0.963 ± 0.325
**Leucine/Isoleucine**	2.28	130.087	331.043 ± 99.910	447.247 ± 261.525	598.998 ± 134.451
**Lysine**	1.08	145.098	9.561 ± 3.006	9.644 ± 5.697	11.955 ± 3.318
**Malate**	12.83	133.014	515.653 ± 103.783	565.405 ± 316.000	559.104 ± 129.501
**Maleic acid**	12.64	115.004	18.422 ± 41.707	37.815 ± 57.219	29.445 ± 43.740
**Methionine**	1.84	148.044	42.949 ± 90.996	3.221 ± 7.768	22.233 ± 39.612
**N-Acetyl-glucosamine-1/6-phosphate**	7.46	300.049	26.028 ± 11.664	12.325 ± 14.698	4.364 ± 4.343

**N-Acetyl-glutamate**	13.54	188.057	8.202 ± 2.459	6.421 ± 4.439	7.113 ± 2.399
**N-Acetyl-glutamine**	7.27	187.073	18.189 ± 4.234	13.132 ± 7.308	16.463 ± 4.225
**N-Acetyl-L-ornithine**	1.39	173.093	4.468 ± 3.332	8.471 ± 7.777	7.353 ± 6.206
**N-Carbamoyl-L-aspartate**	12.62	175.036	69.745 ± 29.368	44.211 ± 46.345	56.354 ± 28.786
**Nicotinate**	11.18	122.025	990.397 ± 170.588	768.613 ± 416.849	770.809 ± 177.304
**O-Acetyl-serine**	1.29	146.046	0.381 ± 0.165	0.258 ± 0.123	0.371 ± 0.177
**Ornithine**	1.08	131.083	11.459 ± 16.599	6.796 ± 9.329	19.839 ± 21.226
**Orotate**	8.38	155.01	75.916 ± 39.050	48.434 ± 67.192	73.572 ± 71.077
**Oxaloacetate**	13.75	130.999	0.307 ± 0.163	0.315 ± 0.167	0.262 ± 0.043
**p-Aminobenzoate**	8.92	136.041	0.313 ± 0.092	0.275 ± 0.141	0.242 ± 0.080
**Pantothenate**	11.23	218.103	584.057 ± 268.080	580.465 ± 404.481	825.544 ± 109.319
**Phenyllactic acid**	14.9	165.056	39.075 ± 7.487	37.004 ± 23.077	31.149 ± 26.787
**Phenylpyruvate**	15.32	163.04	0.244 ± 0.158	0.148 ± 0.039	0.181 ± 0.112

**Phosphoenolpyruvate**	13.94	166.975	0.887 ± 0.709	0.357 ± 0.310	0.175 ± 0.093
**Pipecolic Acid**	1.35	128.072	3.557 ± 4.970	0.265 ± 0.116	6.537 ± 3.618
**Prephenate**	14.26	225.041	1.325 ± 0.621	1.225 ± 0.517	0.732 ± 0.170
**Pro-Glu (PE)**	4.39	243.099	0.942 ± 0.348	1.034 ± 0.568	1.102 ± 0.497
**Proline**	1.33	114.056	56.594 ± 21.315	63.786 ± 44.100	83.568 ± 20.203
**Pyridoxamine**	7.91	167.083	0.263 ± 0.064	0.237 ± 0.103	0.179 ± 0.056
**Pyridoxine**	1.8	168.067	0.327 ± 0.116	0.298 ± 0.137	0.461 ± 0.409
**Pyroglutamic acid**	7.31	128.035	387.699 ± 89.029	348.902 ± 184.583	396.602 ± 79.375
**Pyruvate**	8.64	87.009	8.318 ± 4.570	3.841 ± 1.969	6.021 ± 3.527
**Quinolinate**	13.74	166.015	9.921 ± 2.104	7.942 ± 4.117	8.360 ± 0.613
**Riboflavin**	12.34	375.132	0.475 ± 0.127	0.512 ± 0.396	0.649 ± 0.169
**Ribose**	1.31	149.046	42.881 ± 10.735	33.906 ± 20.169	44.919 ± 23.419
**Ribose-5-phosphate**	7.31	229.012	4.023 ± 3.453	2.571 ± 2.316	0.950 ± 0.442

**Ribulose-5-phosphate**	7.77	229.012	7.602 ± 7.372	4.714 ± 5.985	1.484 ± 0.795
**Ser-Asp (SD)**	3.75	219.062	13.009 ± 1.916	13.488 ± 7.560	12.368 ± 3.926
**Ser-Leu/Ile-Asp (SID)**	10.66	332.147	1.082 ± 0.343	0.967 ± 0.529	1.141 ± 0.431
**Serine**	1.2	104.035	33.773 ± 7.937	42.058 ± 25.032	53.339 ± 13.062
**sn-Glycerol-3-phosphate**	7.47	171.007	64.652 ± 30.105	55.849 ± 72.702	25.354 ± 16.886
**Succinate/Methylmalonic acid**	12.02	117.019	1993.173 ± 496.461	1554.611 ± 795.600	1610.725 ± 257.754
**Taurine**	1.24	124.007	499.395 ± 210.699	447.431 ± 258.645	401.456 ± 181.408
**Thiamine**	1.14	263.097	0.645 ± 0.182	0.692 ± 0.480	0.818 ± 0.289
**Threonine/Homoserine**	1.25	118.051	60.195 ± 12.415	59.959 ± 32.215	85.893 ± 11.075
**Trehalose-6-Phosphate**	6.9	421.076	0.151 ± 0.148	0.106 ± 0.117	0.041 ± 0.043
**Trehalose/Sucrose**	1.31	341.109	9.632 ± 8.843	6.401 ± 8.652	6.939 ± 5.507
**Tryptophan**	7.86	203.083	105.141 ± 26.958	128.268 ± 73.362	167.803 ± 39.143
**UDP**	13.67	402.996	0.241 ± 0.084	0.167 ± 0.158	0.124 ± 0.078

**UDP-D-glucose**	13.27	565.048	0.153 ± 0.078	0.068 ± 0.055	0.057 ± 0.057
**UDP-N-acetyl-glucosamine**	13.3	606.075	0.121 ± 0.068	0.037 ± 0.030	0.051 ± 0.054
**UMP**	10.29	323.029	5.581 ± 2.050	4.229 ± 2.332	4.311 ± 2.162
**Uracil**	1.58	111.02	0.473 ± 0.154	0.480 ± 0.226	0.420 ± 0.198
**Uracil**	1.58	111.02	0.473 ± 0.154	0.480 ± 0.226	0.420 ± 0.198
**Uric acid**	6.32	167.021	13.572 ± 5.723	10.727 ± 10.272	16.075 ± 6.733
**Uric acid**	6.32	167.021	13.572 ± 5.723	10.727 ± 10.272	16.075 ± 6.733
**Uridine**	1.67	243.062	26.297 ± 28.620	21.886 ± 34.102	14.952 ± 12.536
**Uridine**	1.67	243.062	26.297 ± 28.620	21.886 ± 34.102	14.952 ± 12.536
**Val-Asp (VD)**	6.11	231.099	2.237 ± 0.443	1.538 ± 0.814	2.840 ± 1.152
**Val-Asp (VD)**	6.11	231.099	2.237 ± 0.443	1.538 ± 0.814	2.840 ± 1.152
**Valine/5-Aminopentanoic acid**	1.14	116.072	10.610 ± 2.374	9.065 ± 4.484	17.798 ± 13.838
**Valine/5-Aminopentanoic acid**	1.14	116.072	10.610 ± 2.374	9.065 ± 4.484	17.798 ± 13.838

**Xanthine**	2.51	151.026	30.000 ± 8.013	14.655 ± 9.706	21.140 ± 9.894
**Xanthine**	2.51	151.026	30.000 ± 8.013	14.655 ± 9.706	21.140 ± 9.894
**Xanthosine**	7.79	283.069	55.037 ± 12.349	30.417 ± 24.522	26.537 ± 15.379
**Xanthosine**	7.79	283.069	55.037 ± 12.349	30.417 ± 24.522	26.537 ± 15.379
**Xanthosine-5-phosphate**	12.9	363.035	0.733 ± 0.403	0.358 ± 0.311	0.271 ± 0.219
**Xanthosine-5-phosphate**	12.9	363.035	0.733 ± 0.403	0.358 ± 0.311	0.271 ± 0.219
**Xanthurenic acid**	13.91	204.03	1.406 ± 0.468	1.364 ± 0.887	1.977 ± 0.759
**Xanthurenic acid**	13.91	204.03	1.406 ± 0.468	1.364 ± 0.887	1.977 ± 0.759

Supplemental Table S3: Normalized Metabolite Table for Unidentified Metabolites

A list of all the M/Z and retention time values for the top 200 unidentified metabolites after LC-MS analysis.

Average Rt(min)	Average Mz	Average Control	Average 5 µg/kg TCDF	Average 24 µg/kg TCDF	Average Rt(min)
**1.08**	942.9412	0.249 ± 0.066	0.104 ± 0.123	0.102 ± 0.047	**1.08**
**1.7**	272.0461	0.144 ± 0.029	0.076 ± 0.045	0.072 ± 0.025	**1.7**
**2.19**	399.273	0.083 ± 0.023	0.061 ± 0.041	0.027 ± 0.022	**2.19**
**2.28**	129.8757	0.248 ± 0.130	0.354 ± 0.233	0.498 ± 0.058	**2.28**
**2.28**	430.2238	0.143 ± 0.050	0.227 ± 0.154	0.352 ± 0.109	**2.28**
**2.28**	261.182	6.738 ± 3.223	12.363 ± 8.777	19.464 ± 6.641	**2.28**
**2.45**	263.1861	0.139 ± 0.088	0.231 ± 0.229	0.383 ± 0.058	**2.45**
**2.45**	449.143	0.313 ± 0.116	0.163 ± 0.173	0.084 ± 0.070	**2.45**
**2.46**	261.182	22.499 ± 9.914	35.772 ± 23.733	51.273 ± 13.297	**2.46**
**2.46**	261.139	0.057 ± 0.063	0.169 ± 0.155	0.205 ± 0.061	**2.46**
**2.47**	160.0979	0.352 ± 0.154	0.490 ± 0.305	0.802 ± 0.204	**2.47**
**2.5**	218.0403	0.563 ± 0.111	0.849 ± 0.559	0.967 ± 0.205	**2.5**
**2.51**	359.1252	1.620 ± 0.695	2.412 ± 1.559	3.069 ± 0.509	**2.51**
**2.54**	247.0935	0.297 ± 0.078	0.138 ± 0.081	0.090 ± 0.049	**2.54**
**3.31**	389.2048	0.112 ± 0.022	0.191 ± 0.135	0.237 ± 0.049	**3.31**
**3.48**	193.0368	0.025 ± 0.018	0.060 ± 0.021	0.067 ± 0.016	**3.48**
**3.98**	446.2258	0.126 ± 0.048	0.263 ± 0.193	0.258 ± 0.049	**3.98**
**4**	405.1995	0.209 ± 0.031	0.111 ± 0.072	0.099 ± 0.057	**4**
**4.04**	415.1088	0.054 ± 0.012	0.075 ± 0.048	0.092 ± 0.018	**4.04**

**4.19**	167.0462	0.050 ± 0.035	0.212 ± 0.110	0.340 ± 0.151	**4.19**
**4.8**	224.0349	0.018 ± 0.007	0.086 ± 0.048	0.127 ± 0.020	**4.8**
**4.94**	583.1288	0.096 ± 0.016	0.051 ± 0.028	0.042 ± 0.024	**4.94**
**5.17**	300.1201	0.219 ± 0.054	0.279 ± 0.211	0.470 ± 0.050	**5.17**
**5.19**	329.151	23.313 ± 11.468	39.268 ± 27.299	59.007 ± 13.389	**5.19**
**5.19**	202.0455	0.278 ± 0.073	0.439 ± 0.277	0.610 ± 0.149	**5.19**
**5.19**	200.0484	0.833 ± 0.213	1.308 ± 0.888	1.820 ± 0.456	**5.19**
**5.19**	214.0722	0.821 ± 0.305	1.174 ± 0.735	1.673 ± 0.339	**5.19**
**5.19**	180.0666	0.953 ± 0.336	1.333 ± 0.833	1.817 ± 0.352	**5.19**
**5.2**	163.7721	0.421 ± 0.193	0.573 ± 0.347	0.859 ± 0.087	**5.2**
**5.2**	260.0597	0.682 ± 0.136	0.902 ± 0.532	1.152 ± 0.220	**5.2**
**6.12**	203.9364	0.055 ± 0.011	0.034 ± 0.030	0.027 ± 0.011	**6.12**
**6.23**	149.0456	2.369 ± 0.534	1.805 ± 1.059	1.250 ± 0.367	**6.23**
**6.64**	334.1261	0.748 ± 0.119	0.982 ± 0.529	1.151 ± 0.183	**6.64**
**6.84**	137.9867	0.957 ± 0.093	0.519 ± 0.289	0.558 ± 0.146	**6.84**
**6.9**	382.9906	0.006 ± 0.003	0.015 ± 0.013	0.027 ± 0.011	**6.9**
**6.91**	285.0134	0.104 ± 0.023	0.151 ± 0.102	0.267 ± 0.086	**6.91**
**6.92**	125.0357	0.246 ± 0.091	0.399 ± 0.220	0.706 ± 0.201	**6.92**
**7**	358.0121	0.088 ± 0.030	0.038 ± 0.015	0.026 ± 0.010	**7**
**7.07**	148.8638	0.032 ± 0.007	0.039 ± 0.023	0.050 ± 0.008	**7.07**
**7.14**	260.0347	4.535 ± 0.758	2.478 ± 1.399	2.138 ± 0.695	**7.14**
**7.15**	180.007	0.260 ± 0.093	0.702 ± 0.569	1.238 ± 0.458	**7.15**
**7.15**	200.0146	0.065 ± 0.023	0.146 ± 0.093	0.241 ± 0.081	**7.15**
**7.15**	182.0041	0.081 ± 0.027	0.204 ± 0.166	0.408 ± 0.167	**7.15**
**7.16**	144.0302	3.364 ± 1.024	7.641 ± 5.667	13.703 ± 5.042	**7.16**

**7.17**	100.0405	0.896 ± 0.209	1.916 ± 1.207	3.342 ± 1.239	**7.17**
**7.5**	222.9992	0.562 ± 0.073	0.462 ± 0.245	0.382 ± 0.063	**7.5**
**7.5**	283.0236	0.364 ± 0.042	0.271 ± 0.152	0.241 ± 0.056	**7.5**
**7.55**	309.0498	0.242 ± 0.045	0.143 ± 0.089	0.086 ± 0.035	**7.55**
**7.55**	336.0262	0.126 ± 0.056	0.048 ± 0.059	0.011 ± 0.015	**7.55**
**7.59**	297.0496	0.239 ± 0.041	0.128 ± 0.070	0.105 ± 0.039	**7.59**
**7.61**	249.0188	0.381 ± 0.161	1.127 ± 0.603	1.744 ± 0.568	**7.61**
**7.64**	391.0655	0.140 ± 0.034	0.068 ± 0.045	0.048 ± 0.027	**7.64**
**7.64**	272.0544	0.485 ± 0.123	0.250 ± 0.150	0.197 ± 0.074	**7.64**
**7.64**	283.034	0.451 ± 0.149	0.241 ± 0.170	0.142 ± 0.063	**7.64**
**7.64**	355.0554	0.194 ± 0.051	0.127 ± 0.071	0.099 ± 0.022	**7.64**
**7.64**	412.0421	1.052 ± 0.583	0.296 ± 0.376	0.066 ± 0.060	**7.64**
**7.65**	224.9824	0.392 ± 0.116	0.174 ± 0.147	0.074 ± 0.042	**7.65**
**7.65**	372.0706	0.232 ± 0.046	0.125 ± 0.082	0.084 ± 0.039	**7.65**
**7.65**	271.0592	0.641 ± 0.170	0.274 ± 0.146	0.245 ± 0.059	**7.65**
**7.65**	333.0598	0.316 ± 0.082	0.207 ± 0.205	0.110 ± 0.053	**7.65**
**7.65**	315.0491	0.636 ± 0.153	0.379 ± 0.232	0.284 ± 0.098	**7.65**
**7.65**	328.0807	0.147 ± 0.040	0.099 ± 0.053	0.067 ± 0.014	**7.65**
**7.65**	326.0651	0.854 ± 0.223	0.491 ± 0.299	0.372 ± 0.169	**7.65**
**7.66**	320.9551	0.267 ± 0.039	0.103 ± 0.115	0.042 ± 0.042	**7.66**
**7.66**	190.9518	2.902 ± 0.616	1.319 ± 1.135	0.745 ± 0.388	**7.66**
**7.66**	271.023	0.259 ± 0.054	0.143 ± 0.090	0.102 ± 0.028	**7.66**
**7.66**	317.0649	0.180 ± 0.035	0.115 ± 0.087	0.064 ± 0.028	**7.66**
**7.66**	112.9896	0.906 ± 0.125	0.744 ± 0.246	0.428 ± 0.138	**7.66**
**7.66**	222.978	24.886 ± 7.096	11.044 ± 9.897	5.718 ± 2.921	**7.66**

**7.66**	110.9853	103.123 ± 15.801	64.273 ± 40.237	47.154 ± 16.060	**7.66**
**7.66**	148.959	0.489 ± 0.064	0.328 ± 0.212	0.259 ± 0.072	**7.66**
**7.66**	361.0547	0.290 ± 0.048	0.205 ± 0.119	0.150 ± 0.044	**7.66**
**7.66**	312.0494	0.243 ± 0.042	0.143 ± 0.082	0.126 ± 0.042	**7.66**
**7.66**	208.9623	3.531 ± 0.881	1.918 ± 1.368	1.395 ± 0.632	**7.66**
**7.66**	386.0862	0.079 ± 0.021	0.041 ± 0.026	0.028 ± 0.016	**7.66**
**7.66**	286.0337	0.357 ± 0.070	0.234 ± 0.139	0.183 ± 0.076	**7.66**
**7.67**	384.0819	0.147 ± 0.022	0.102 ± 0.073	0.067 ± 0.018	**7.67**
**7.67**	356.9529	0.854 ± 0.177	0.443 ± 0.451	0.213 ± 0.155	**7.67**
**7.67**	411.0815	0.165 ± 0.030	0.087 ± 0.056	0.068 ± 0.020	**7.67**
**7.67**	415.113	0.088 ± 0.018	0.074 ± 0.051	0.033 ± 0.011	**7.67**
**7.67**	222.9441	0.162 ± 0.053	0.065 ± 0.072	0.021 ± 0.023	**7.67**
**7.67**	430.076	0.903 ± 0.187	0.522 ± 0.323	0.365 ± 0.123	**7.67**
**7.67**	244.9599	3.314 ± 0.579	1.991 ± 1.475	1.288 ± 0.622	**7.67**
**7.67**	468.9453	0.236 ± 0.090	0.083 ± 0.095	0.021 ± 0.027	**7.67**
**7.67**	480.0916	0.056 ± 0.009	0.039 ± 0.040	0.022 ± 0.012	**7.67**
**7.67**	372.9178	0.121 ± 0.031	0.069 ± 0.067	0.026 ± 0.031	**7.67**
**7.67**	154.9924	4.060 ± 0.643	2.624 ± 1.477	2.075 ± 0.724	**7.67**
**7.67**	136.9817	0.258 ± 0.046	0.174 ± 0.107	0.132 ± 0.043	**7.67**
**7.67**	334.9709	1.505 ± 0.662	0.533 ± 0.616	0.181 ± 0.125	**7.67**
**7.67**	212.9338	0.189 ± 0.031	0.119 ± 0.087	0.081 ± 0.046	**7.67**
**7.67**	153.996	0.956 ± 0.165	0.623 ± 0.379	0.491 ± 0.178	**7.67**
**7.67**	454.9299	0.049 ± 0.022	0.020 ± 0.042	0.007 ± 0.011	**7.67**
**7.67**	313.0447	3.489 ± 1.074	1.824 ± 1.153	1.460 ± 0.469	**7.67**
**7.67**	260.9338	0.055 ± 0.023	0.018 ± 0.022	0.015 ± 0.005	**7.67**

**7.68**	146.962	1.541 ± 0.217	1.010 ± 0.616	0.756 ± 0.217	**7.68**
**7.68**	369.071	0.566 ± 0.128	0.345 ± 0.231	0.206 ± 0.067	**7.68**
**7.68**	316.0806	1.008 ± 0.235	0.491 ± 0.372	0.342 ± 0.135	**7.68**
**7.68**	401.1335	1.842 ± 0.325	1.109 ± 0.693	0.891 ± 0.271	**7.68**
**7.68**	511.1451	0.077 ± 0.020	0.034 ± 0.026	0.029 ± 0.012	**7.68**
**7.68**	330.06	0.622 ± 0.151	0.358 ± 0.223	0.260 ± 0.092	**7.68**
**7.69**	314.065	0.279 ± 0.086	0.156 ± 0.105	0.108 ± 0.026	**7.69**
**7.69**	245.0433	1.802 ± 0.698	1.201 ± 1.915	0.511 ± 0.175	**7.69**
**7.7**	324.0494	0.127 ± 0.016	0.091 ± 0.062	0.063 ± 0.024	**7.7**
**7.7**	339.0603	0.723 ± 0.186	0.442 ± 0.314	0.329 ± 0.085	**7.7**
**7.72**	247.0309	0.056 ± 0.023	0.414 ± 0.217	0.573 ± 0.124	**7.72**
**7.72**	245.0351	1.628 ± 0.741	10.011 ± 5.152	12.961 ± 3.063	**7.72**
**7.72**	356.0394	0.434 ± 0.094	0.245 ± 0.172	0.218 ± 0.039	**7.72**
**7.76**	395.0614	0.517 ± 0.231	0.168 ± 0.163	0.107 ± 0.066	**7.76**
**7.8**	585.1805	0.083 ± 0.018	0.028 ± 0.032	0.021 ± 0.022	**7.8**
**7.8**	283.1186	0.313 ± 0.057	0.127 ± 0.101	0.128 ± 0.066	**7.8**
**7.8**	319.0453	1.029 ± 0.242	0.553 ± 0.383	0.424 ± 0.227	**7.8**
**7.82**	222.0355	0.542 ± 0.109	0.345 ± 0.192	0.330 ± 0.063	**7.82**
**7.83**	450.0941	1.253 ± 0.300	0.756 ± 0.583	0.441 ± 0.339	**7.83**
**8.05**	182.0936	0.152 ± 0.020	0.254 ± 0.145	0.245 ± 0.028	**8.05**
**8.32**	144.0303	1.619 ± 0.438	1.830 ± 1.267	3.495 ± 0.787	**8.32**
**8.41**	241.9985	0.017 ± 0.007	0.038 ± 0.033	0.064 ± 0.020	**8.41**
**8.41**	99.0089	0.714 ± 0.210	1.406 ± 0.727	1.514 ± 0.341	**8.41**
**8.56**	168.0304	3.185 ± 0.609	1.891 ± 1.351	1.376 ± 0.749	**8.56**
**8.7**	303.0331	0.495 ± 0.099	0.353 ± 0.319	0.254 ± 0.092	**8.7**

**8.96**	178.0259	0.544 ± 0.127	0.299 ± 0.167	0.273 ± 0.082	**8.96**
**9.05**	342.1674	1.138 ± 0.214	1.591 ± 1.023	1.927 ± 0.383	**9.05**
**9.14**	584.1319	0.062 ± 0.025	0.041 ± 0.027	0.014 ± 0.010	**9.14**
**9.3**	323.1617	0.049 ± 0.017	0.075 ± 0.060	0.229 ± 0.058	**9.3**
**9.34**	221.0239	1.969 ± 0.167	1.307 ± 0.688	1.134 ± 0.285	**9.34**
**9.34**	629.1952	0.015 ± 0.004	0.018 ± 0.012	0.006 ± 0.003	**9.34**
**9.52**	260.0235	0.090 ± 0.029	0.221 ± 0.114	0.246 ± 0.072	**9.52**
**9.52**	173.0027	0.181 ± 0.010	0.144 ± 0.034	0.119 ± 0.032	**9.52**
**9.67**	341.0759	1.605 ± 0.648	0.669 ± 0.798	0.187 ± 0.151	**9.67**
**9.93**	421.0757	0.046 ± 0.017	0.029 ± 0.033	0.009 ± 0.008	**9.93**
**10.06**	246.0191	0.200 ± 0.024	0.120 ± 0.070	0.113 ± 0.042	**10.06**
**10.19**	294.0597	0.198 ± 0.040	0.107 ± 0.065	0.106 ± 0.033	**10.19**
**10.2**	153.7422	0.162 ± 0.031	0.099 ± 0.067	0.092 ± 0.016	**10.2**
**10.28**	289.0943	0.238 ± 0.032	0.154 ± 0.083	0.144 ± 0.046	**10.28**
**10.4**	311.1729	0.193 ± 0.047	0.150 ± 0.048	0.091 ± 0.032	**10.4**
**10.47**	157.9918	0.293 ± 0.064	0.171 ± 0.072	0.136 ± 0.049	**10.47**
**10.61**	386.067	0.035 ± 0.003	0.022 ± 0.012	0.023 ± 0.006	**10.61**
**10.8**	263.0676	0.421 ± 0.177	1.107 ± 0.713	1.236 ± 0.406	**10.8**
**10.89**	174.0773	0.480 ± 0.080	0.220 ± 0.081	0.183 ± 0.058	**10.89**
**11.05**	196.1093	0.207 ± 0.085	0.690 ± 0.364	0.654 ± 0.182	**11.05**
**11.24**	325.0809	0.831 ± 0.370	0.298 ± 0.337	0.122 ± 0.111	**11.24**
**11.25**	276.0622	0.183 ± 0.032	0.202 ± 0.136	0.296 ± 0.043	**11.25**
**11.26**	344.1331	0.096 ± 0.020	0.114 ± 0.090	0.192 ± 0.039	**11.26**
**11.26**	300.1067	4.210 ± 1.322	4.942 ± 3.490	7.041 ± 1.014	**11.26**
**11.27**	451.2302	0.048 ± 0.027	0.123 ± 0.092	0.149 ± 0.033	**11.27**

**11.27**	328.1382	0.263 ± 0.064	0.286 ± 0.198	0.446 ± 0.074	**11.27**
**11.29**	302.136	3.465 ± 0.373	4.261 ± 2.470	4.804 ± 0.697	**11.29**
**11.37**	351.1566	0.424 ± 0.089	0.245 ± 0.246	0.201 ± 0.067	**11.37**
**11.4**	199.0015	0.433 ± 0.163	0.157 ± 0.085	0.099 ± 0.069	**11.4**
**11.61**	616.2953	0.168 ± 0.072	0.104 ± 0.077	0.031 ± 0.020	**11.61**
**11.9**	263.0345	0.257 ± 0.095	0.828 ± 0.467	1.241 ± 0.375	**11.9**
**11.96**	184.9872	0.318 ± 0.064	0.131 ± 0.099	0.115 ± 0.035	**11.96**
**12.29**	432.1377	0.447 ± 0.116	0.419 ± 0.303	0.242 ± 0.018	**12.29**
**12.46**	258.9919	0.163 ± 0.048	0.050 ± 0.027	0.056 ± 0.026	**12.46**
**12.5**	457.1206	0.229 ± 0.072	0.200 ± 0.110	0.094 ± 0.034	**12.5**
**13.01**	377.0883	0.297 ± 0.059	0.257 ± 0.138	0.152 ± 0.061	**13.01**
**13.02**	256.0036	2.271 ± 0.390	1.031 ± 0.650	0.818 ± 0.255	**13.02**
**13.04**	249.0552	0.502 ± 0.171	1.189 ± 0.601	1.251 ± 0.161	**13.04**
**13.23**	355.028	0.215 ± 0.031	0.124 ± 0.075	0.117 ± 0.045	**13.23**
**13.46**	300.9809	0.165 ± 0.109	0.498 ± 0.279	0.665 ± 0.220	**13.46**
**13.46**	222.0538	0.251 ± 0.124	0.064 ± 0.093	0.014 ± 0.013	**13.46**
**13.76**	250.9982	0.539 ± 0.040	0.294 ± 0.204	0.351 ± 0.100	**13.76**
**14.08**	263.0424	0.876 ± 0.105	0.532 ± 0.316	0.493 ± 0.198	**14.08**
**14.1**	222.008	2.180 ± 0.646	4.001 ± 2.043	4.733 ± 0.669	**14.1**
**14.1**	433.2442	0.071 ± 0.019	0.042 ± 0.039	0.023 ± 0.020	**14.1**
**14.12**	289.998	0.029 ± 0.009	0.037 ± 0.024	0.059 ± 0.013	**14.12**
**14.14**	292.0831	0.933 ± 0.228	1.512 ± 0.766	1.608 ± 0.310	**14.14**
**14.37**	434.092	0.010 ± 0.009	0.078 ± 0.044	0.154 ± 0.063	**14.37**
**14.37**	263.0709	0.656 ± 0.261	1.084 ± 0.542	1.146 ± 0.120	**14.37**
**14.61**	689.3745	0.233 ± 0.065	0.148 ± 0.168	0.043 ± 0.085	**14.61**

**14.73**	816.2764	0.277 ± 0.056	0.202 ± 0.212	0.083 ± 0.064	**14.73**
**14.77**	648.2936	0.081 ± 0.029	0.059 ± 0.050	0.020 ± 0.010	**14.77**
**14.79**	211.0614	1.443 ± 0.215	0.802 ± 0.398	0.647 ± 0.317	**14.79**
**14.85**	407.1328	2.067 ± 0.438	1.663 ± 1.567	0.784 ± 0.559	**14.85**
**14.86**	815.2733	0.814 ± 0.159	0.646 ± 0.631	0.260 ± 0.190	**14.86**
**14.87**	387.1094	0.491 ± 0.268	0.277 ± 0.212	1.387 ± 0.448	**14.87**
**14.99**	467.0661	0.067 ± 0.031	0.039 ± 0.022	0.173 ± 0.055	**14.99**
**15.07**	331.083	1.297 ± 0.244	0.836 ± 0.449	0.689 ± 0.230	**15.07**
**15.07**	607.1677	0.250 ± 0.135	0.027 ± 0.038	0.013 ± 0.015	**15.07**
**15.15**	524.9996	0.008 ± 0.011	0.091 ± 0.062	0.075 ± 0.036	**15.15**
**15.22**	297.1712	0.542 ± 0.131	0.231 ± 0.159	0.140 ± 0.036	**15.22**
**15.45**	380.9382	0.003 ± 0.005	0.028 ± 0.023	0.045 ± 0.014	**15.45**
**15.51**	359.0815	0.994 ± 0.132	0.564 ± 0.313	0.489 ± 0.178	**15.51**
**16.13**	285.0408	0.573 ± 0.336	0.815 ± 0.418	1.525 ± 0.401	**16.13**
**16.58**	588.7601	0.451 ± 0.139	0.335 ± 0.278	0.103 ± 0.095	**16.58**
**16.6**	365.1611	0.404 ± 0.099	0.234 ± 0.125	0.204 ± 0.045	**16.6**
**16.64**	589.7625	0.076 ± 0.029	0.056 ± 0.052	0.010 ± 0.009	**16.64**
**17.26**	379.1766	1.869 ± 0.420	1.155 ± 0.615	1.036 ± 0.181	**17.26**
**17.28**	293.1397	0.165 ± 0.061	0.373 ± 0.088	0.333 ± 0.067	**17.28**
**17.38**	299.1504	0.082 ± 0.042	0.216 ± 0.063	0.348 ± 0.089	**17.38**
**17.38**	365.1609	0.197 ± 0.038	0.094 ± 0.051	0.085 ± 0.021	**17.38**
**17.4**	303.1453	0.360 ± 0.170	0.067 ± 0.034	0.055 ± 0.027	**17.4**
**17.74**	321.0522	0.002 ± 0.005	0.072 ± 0.045	0.129 ± 0.022	**17.74**
**17.82**	349.1662	1.105 ± 0.223	0.890 ± 0.828	0.464 ± 0.246	**17.82**
**18.64**	345.1381	0.274 ± 0.064	0.384 ± 0.219	0.542 ± 0.119	**18.64**

**19.13**	335.187	1.411 ± 0.391	1.761 ± 0.708	2.644 ± 0.584	**19.13**
**19.14**	390.1266	0.000 ± 0.000	0.404 ± 0.296	0.435 ± 0.130	**19.14**
**19.14**	194.5594	0.006 ± 0.009	0.166 ± 0.108	0.160 ± 0.072	**19.14**
**19.15**	391.1108	0.004 ± 0.009	0.266 ± 0.240	0.252 ± 0.123	**19.15**
**19.37**	447.2394	0.329 ± 0.039	0.221 ± 0.122	0.178 ± 0.068	**19.37**
**19.52**	331.1323	0.183 ± 0.042	0.072 ± 0.061	0.061 ± 0.043	**19.52**

Supplemental Table S4: **Primer Sequences for qPCR Analysis of Tight Junction Proteins**

Gene	FP	RP	Gene Name

mOcln	CGG CCG CCA AGG TTC GCT TA	CCT GGG GGC GAC GTC CAT TT	Occludin
mTjp1 (Zo-1)	CTT TCG CCT GAA ACA ACC CC	CTG TAC ACC TTT GCT GGG TCT	Zonula occludens 1
mTjp2 (Zo-2)	TCG TCG GGT GAC CCT AAA AC	ACA GTT GGC TCC AAC AAG GT	Zonula occludens 2
mGapdh	CCT CGT CCC GTA GAC AAA ATG	TGA AGG GGT CGT TGA TGG C	Glyceraldehyde 3-phosphate dehydrogenase

Supplemental Figure S1: Bacteroidetes to Firmicutes ratio

A bar chart representing the Bacteroidetes to Firmicutes ratio between control and 5 µg/kg BW TCDF exposure and control and 24 µg/kg BW TCDF exposure.

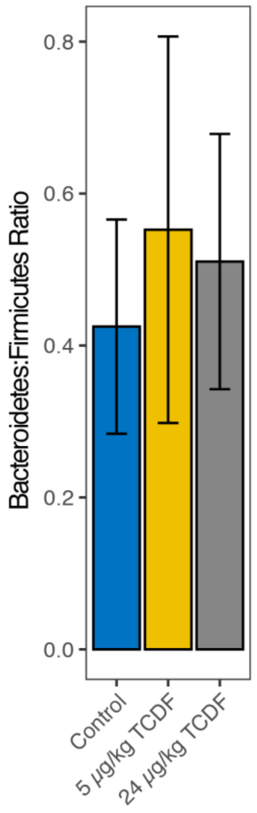

Supplemental Figure S2: Top 50 significant KEGG enzymes and COGs after 24 µg/kg BW TCDF exposure TCDF exposure

A heat map of the Z-scores of the top 50 significantly different enzymes after 24 μg/kg BW TCDF exposure.

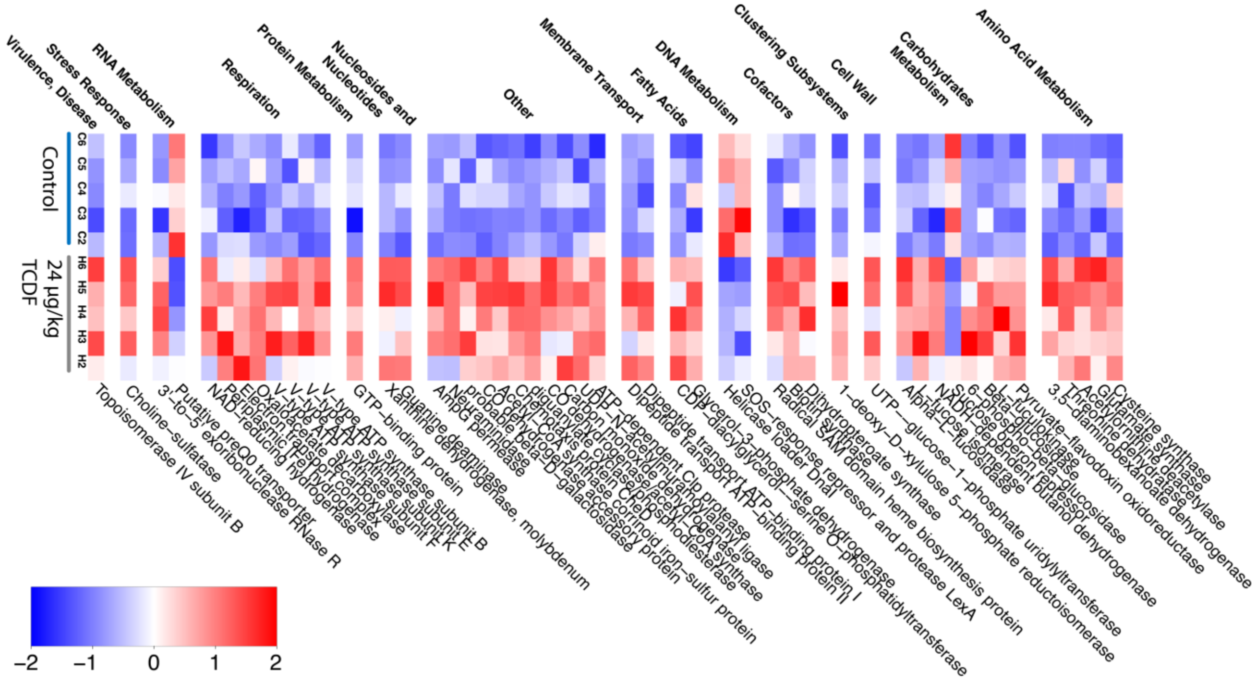

Supplement: Supplementary file 1 [file metabolites-10-00001-s001.pdf]
